# *Colpodella* sp. (ATCC 50594) Life Cycle: Myzocytosis and Possible Links to the Origin of Intracellular Parasitism

**DOI:** 10.3390/tropicalmed6030127

**Published:** 2021-07-11

**Authors:** Troy A. Getty, John W. Peterson, Hisashi Fujioka, Aidan M. Walsh, Tobili Y. Sam-Yellowe

**Affiliations:** 1 Department of Biological, Geological and Environmental Sciences, Cleveland State University, Cleveland, OH 44115, USA; t.getty@vikes.csuohio.edu (T.A.G.); aidanwalshcreativestudios@gmail.com (A.M.W.); 2 Cleveland Clinic Lerner Research Institute, Cleveland, OH 44195, USA; PETERSJ@ccf.org; 3 Cryo-EM Core, Cleveland Center for Membrane and Structural Biology, Case Western Reserve University, Cleveland, OH 44106, USA; hxf3@case.edu

**Keywords:** apicomplexa, *Colpodella* apical complex organelles, *Colpodella* proteins, *Colpodella* cysts, *Colpodella* species, *Colpodella* ultrastructure, life cycle, myzocytosis, trichrome stain

## Abstract

*Colpodella* species are free living bi-flagellated protists that prey on algae and bodonids in a process known as myzocytosis. *Colpodella* species are phylogenetically related to Apicomplexa. We investigated the life cycle of *Colpodella* sp. (ATCC 50594) to understand the timing, duration and the transition stages of *Colpodella* sp. (ATCC 50594). Sam-Yellowe’s trichrome stains for light microscopy, confocal and differential interference contrast (DIC) microscopy was performed to identify cell morphology and determine cross reactivity of *Plasmodium* species and *Toxoplasma gondii* specific antibodies against *Colpodella* sp. (ATCC 50594) proteins. The ultrastructure of *Colpodella* sp. (ATCC 50594) was investigated by transmission electron microscopy (TEM). The duration of *Colpodella* sp. (ATCC 50594) life cycle is thirty-six hours. *Colpodella* sp. (ATCC 50594) were most active between 20–28 h. Myzocytosis is initiated by attachment of the *Colpodella* sp. (ATCC 50594) pseudo-conoid to the cell surface of *Parabodo caudatus,* followed by an expansion of microtubules at the attachment site and aspiration of the prey’s cytoplasmic contents. A pre-cyst formed at the conclusion of feeding differentiates into a transient or resting cyst. Both DIC and TEM microscopy identified asynchronous and asymmetric mitosis in *Colpodella* sp. (ATCC 50594) cysts. Knowledge of the life cycle and stages of *Colpodella* sp. (ATCC 50594) will provide insights into the development of intracellular parasitism among the apicomplexa.

## 1. Introduction

*Colpodella* species are free-living terrestrial, fresh water or marine predators that feed on protists and algae. Trophozoite and cyst stages have been described in the life cycles of *Colpodella* species [1]. Trophozoites have a cone shaped microtubular structure which forms the pseudo-conoid contained in the rostrum, used for feeding [1,2]. *Colpodella* species possess a pellicle and apical complex organelles, like pathogenic apicomplexans, that include rhoptries, micronemes, pseudo-conoid, polar rings and microtubules, which facilitate predation. Trophozoites possess hetero-dynamic flagella that originate from separate flagella pockets and possess transversal plates in the transitional zone. A thin wall cylinder lies over the transversal plate. [2,3]. Some *Colpodella* species possess tricho-cysts which are organelles that are ejected in response to stimuli [3].

The mechanisms of encystation, excystation and transformation of life cycle stages is unknown among colpodellids and has not been described in culture or in the environment. The life cycles of *Colpodella vorax*, *C. unguis*, *C. turpis* and *C. pugnax* have been reported [1,3]. However, its duration and the timing of stage transformations were not described in these studies [1,3]. Studies aimed at investigating the biology of *Colpodella* species would benefit from knowing when specific life cycle stages occur in culture to facilitate isolation of specific stages and subcellular organelles. Feeding is initiated when the predator attaches onto the prey using its rostrum where the apical complex organelles are located. A tubular tether forms between the predator and prey and is used for aspirating cytoplasmic contents from the prey. The prey’s contents are aspirated into a posterior food vacuole which enlarges during and after feeding. This type of feeding is known as myzocytosis [3,4]. The posterior food vacuole differentiates into a cyst along with a remnant cytoplasm and the nucleus, following the loss of the anterior end of the trophozoite. The cyst then divides into two, then four cells, that eventually excyst to release trophozoites so the cycle may continue [1,3]. Immature trophozoites egress from the cyst and move rapidly in a characteristic oscillatory motion in search of prey [3]. In previous studies we showed that *Colpodella* sp. (ATCC 50594) forms cysts that divide to contain more than four juveniles. Cysts containing uneven number of juveniles, as low as three to seven juveniles, were observed [5]. Division within the cysts of *Colpodella* sp. (ATCC 50594) were found to be asymmetric and asynchronous, with juveniles having different rates of development within the cysts [5]. 

Single or multiple predators can attach to a single prey on any side. *Colpodella gonderi* and *C. tetrahymenae* are described as ectoparasites and remain attached to their prey for long periods of time [6,7]. *Colpodella tetrahymenae* form cysts with four juveniles following feeding [6]. Among colpodellids, there are cyst forming and non-cyst forming species [1,2,3].

*Colpodella*-like species have been recently reported as potential infectious agents. Two cases of opportunistic human infections believed to be caused by *Colpodella*-like species were reported [8,9]. Infection of red blood cells was reported in the first case [8] and Jiang et al. [9] reported a tick borne *Colpodella* infection. Although polymerase chain reaction (PCR) amplification of blood, cerebrospinal fluid and tick samples identified *Colpodella* sp. DNA, life cycle stages of *Colpodella* species were not identified in host tissue. Furthermore, *Colpodella* species’ DNA was identified from ticks infecting cattle and from raccoons [10,11,12]. Specific life cycle stages were not described in the animal studies. *Colpodella gonderi* and *Colpoda steinii* were identified in the urine of an individual with multiple chronic diseases. Both protists were not found to contribute to urinary infections in the reported case [13]. However, the three reported cases were associated with immunosuppressed human hosts. Through 18S rRNA analysis it was shown that *Colpodella* sp. is phylogenetically related to Apicomplexan parasites such as *Plasmodium* species and the plastid containing *Chromera velia* [14,15]. Phylogenetic analysis shows that *Colpodella* sp. (ATCC 50594) are related to *Colpodella pontica* (renamed *Voromonas pontica*), *Colpodella tetrahymenae* and the pathogenic apicomplexans, *Cryptosporidium serpenti*, *C, muris* and *Toxoplasma gondii* [6,16]. In a previous study, we identified transitional stages of *Colpodella* sp. (ATCC 50594) in the life cycle [5]. However, the duration, transformation and timing of life cycle stages remained unclear. A type of study focused on determining life cycle stage transformations have not been performed with any *Colpodella* species.

In the current study we employed time course experiments to investigate the duration, timing and stage transformations in the life cycle of *Colpodella* sp. (ATCC 50594) in detail. In order to gain insights into how intracellular parasitism developed among the apicomplexans, the life cycle stages involved in myzocytosis, encystation and excystation in *Colpodella* species (ATCC 50594) were investigated. In previous studies, antibodies specific to RhopH3, a rhoptry protein of *Plasmodium* species, were shown to cross react with *Colpodella* sp. (ATCC 50594) [17]. In the current study we investigated the reactivity of antibodies against other rhoptry and microneme proteins of *P. falciparum*. In addition, antibodies against a food vacuole protein of *P. falciparum* and inner membrane complex (IMC) proteins of *Toxoplasma gondii* were also investigated for cross reactivity against *Colpodella* sp. (ATCC 50594) proteins in the different life cycle stages. Our hypothesis is that *Colpodella* sp. (ATCC 50594) has life cycle stages representative of the known cyst forming *Colpodella* species. Recently described trichrome staining protocols facilitated differentiation of cyst stages from predator and prey [5,18].

## 2. Materials and Methods

### 2.1. Hay Medium Cultures

*Colpodella* sp. (ATCC 50594) were maintained in Hay medium as a di-protist culture. Hay medium was bacterized with *Enterobacter aerogenes* in tissue culture flasks as described [19]. Ten mL of culture was maintained in T25 flasks and 30 mL in T75 flasks. *Bodo caudatus* (renamed *Parabodo caudatus* Dujardin) in the di-protist culture served as prey for *Colpodella* sp. (ATCC 50594). Cultures were examined using an inverted microscope to observe different stages of trophozoites and cysts as described [19].

### 2.2. Fixation of Cells

*Colpodella* sp. (ATCC 50594) in di-protist cultures were fixed using 5% formalin as described [18,19]. Briefly, a viable di-protist culture was mixed with equal volume of 10% formalin directly in the culture flask (fixed-in-flight) and incubated for ten minutes at room temperature [19,20]. The fixed cells were scraped gently with a cell scraper and the cells were transferred to a 50 mL centrifuge tube for centrifugation as described previously [19,20]. Following centrifugation, the supernatant was discarded, and the pellets were resuspended in 1× Dulbecco’s phosphate buffered saline (dPBS) and centrifuged. The dPBS supernatant was discarded, and the pellets were resuspended in 100 μL of dPBS. Ten μL of cells was placed on glass slides to prepare smears. The smears were air-dried at room temperature and then used for staining and immunofluorescence assays.

### 2.3. Staining

For light microscopy, formalin fixed cells were stained with Giemsa, Kinyoun’s carbol fucshin (KCF), and Sam-Yellowe’s trichrome stains [5]. Giemsa staining differentiates trophozoites of *Colpodella* sp. (ATCC 50594) and *Parabodo caudatus.* All stained smears were examined under oil immersion at ×1000 magnification and images were captured using an Olympus BX43 compound microscope (Tokyo, Japan) attached to an Infinity HD Lumenera digital camera and Olympus U-TV0.35xc-2 adapter using Infinity HD Capture software.

### 2.4. Time Course Studies of Colpodella sp. (ATCC 50594) Development in Culture

In order to identify life cycle stages of *Colpodella* sp. (ATCC 50594) in Hay medium culture, a time course analysis of *Colpodella* sp. (ATCC 50594) development was performed. Subculture of 500 μL resting cyst stages of *Colpodella* sp. (ATCC 50594) and *P. caudatus* was performed in 10 mL of Hay medium in each T25 flask (nine flasks), to initiate the time course. The initial subculture of cells was collected at T = 0 and then cells were collected every four hours for 36 h (T = 1, T = 2, T = 3, T = 4, T = 5, T = 6, T = 7, T = 8, and T = 9). The first time course experiment was performed in four replicates. Each replicate was performed for 36 h using slide culture chambers for the first two replicates and tissue culture flasks for the second two. The four replicates were performed to determine the reproducibility of the life cycle and stage transitions. Slide culture chambers contained 2 mL cultures in duplicate per slide. Encysted cultures were also collected 5 and 7 days after encystation to identify the morphology of the resting cyst stages. In order to observe cells in the most active period of the life cycle, an additional time course experiment was performed for 40 h. Cells were collected for fixation and staining every four hours up until twenty-two hours. At twenty-two hours cells were collected every hour until 30 h. Then cells were collected for fixation and staining every two hours until 40 h. A final time course experiment was performed to identify the predominant cyst stage of *Colpodella* sp. (ATCC 50594) in resting cultures. Cells were collected every 24 h for eight days and formalin-fixed for staining with Sam-Yellowe’s trichrome staining [18]. Days five, seven and eight cultures were fixed, stained and counted. One hundred cysts on duplicate slides were counted to obtain the percentage of each *Colpodella* sp. (ATCC 50594) life cycle stage present in resting cultures.

### 2.5. Immunofluorescence and Differential Interference Contrast Microscopy

Immunofluorescence assay (IFA) was performed on cells from the di-protist culture fixed using 5% formalin. Formalin fixed cells were permeabilized with 0.1% Triton X-100 then blocked with 3% bovine serum albumin (BSA). After blocking, incubation occurred with primary antibodies specific for antigen. This incubation was followed by three washes with 1× dPBS and then the smears were incubated with species specific secondary antibody conjugated to an Alexa fluorophore. Alexa fluorophores used include Alexa 488 and Alexa 647. Antibodies specific to apical complex organelle proteins were used to identify *Colpodella* sp. (ATCC 50594) proteins as described [17]. Antiserum 686 [21] specific for the rhoptry protein RhopH3 was used for IFAs. Other antibodies used were anti-Py235 [22], Anti-plasmepsin II [23], anti-EBA175 [24], anti-AMA1 [25], anti-IMC3, anti-IMC3 FLR, anti-IMC7 [26,27,28] and anti-RhopH3 full length (FL) [29]. In some experiments, cells were stained with Actin green 488 to detect actin and then reacted with antibodies for immunofluorescence. IFA slides were examined at the imaging core (Learner Research Institute, Cleveland Clinic). Confocal, fluorescent and differential interference contrast (DIC) images were collected using a Leica SP8 True Scanning Confocal (TCS) DM18 inverted microscope (Leica Microsystems, GmbH, Wetzlar, Germany). Stained and confocal images were adjusted to 300 dpi using CYMK color mode, RGB color mode, auto color and auto contrast on Adobe Photoshop (CC). 3D reconstructions of confocal z-stacks were performed using Volocity v.6.3.0 software (Quorum Technologies Inc., Puslinch, ON, Canada).

### 2.6. Transmission Electron Microscopy

An aliquot of *Colpodella* sp. (ATCC 50594) in culture medium was added to an equal volume of 8% paraformaldehyde in 0.1 M cacodylate buffer and spun down for 10 min at 3500 rpm. The cell pellet was fixed with 2.5% glutaraldehyde, 2% paraformaldehyde in 0.1 M cacodylate buffer. The fixation continued in the same fixative solution for a total of 2 h at room temperature. The pellets were thoroughly rinsed in 0.1 M cacodylate buffer, and then postfixed for 2 h in an unbuffered 1:1 mixture of 2% osmium tetroxide and 3% potassium ferrocyanide. After rinsing with distilled water, the specimens were soaked overnight in an acidified solution of 0.25% uranyl acetate. After another rinse in distilled water, they were dehydrated in ascending concentrations of ethanol, passed through propylene oxide, and embedded in an EMbed 812 embedding media (Electron Microscopy Sciences, Hatfield, PA, USA). Thin sections (70 nm) were cut on a RMC MT6000-XL ultramicrotome. These were mounted on T-300 mesh nickel grids (Electron Microscopy Sciences, Hatfield, PA, USA) and then sequentially stained with acidified methanolic uranyl acetate and stable lead staining solution. They were then coated on a Denton DV-401 carbon coater (Denton Vacuum LLC, Moorestown, NJ, USA), and observed in a FEI Tecnai Spirit (T12) transmission electron microscope with a Gatan US4000 4k × 4k CCD.

## 3. Results

### 3.1. General Staining

**Figure 1:**
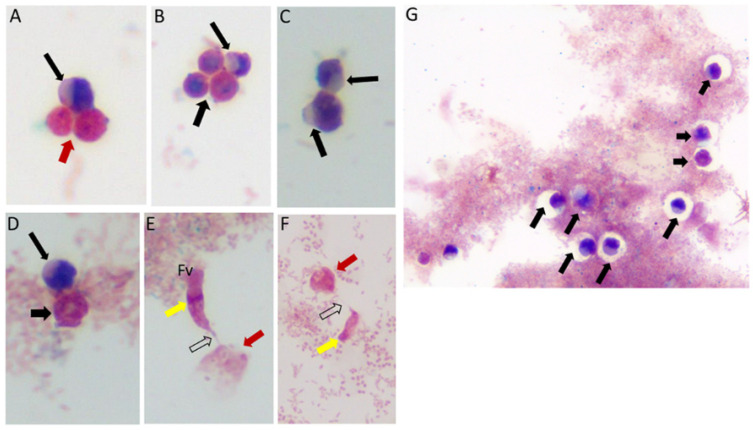
Life cycle stages observed from di-protist cultures of *Colpodella* sp. (ATCC 50594) and *Parabodo caudatus*. Black arrows in panels (**A**–**D**,**G**) depict young demilune cysts of *Colpodella* sp. (ATCC 50594). Black arrowheads show mature cysts, red arrows show cysts of *P. caudatus*. In panels (**E**,**F**) yellow arrows show *Colpodella* sp. (ATCC 50594) trophozoites in myzo-cytosis with *P. caudatus* prey (red arrows). Open arrows identify tubular tether formed between predator and prey. Clear zones separating bacteria from cysts of *Colpodella* sp. (ATCC 50594) were observed, black arrows.

**Figure 2:**
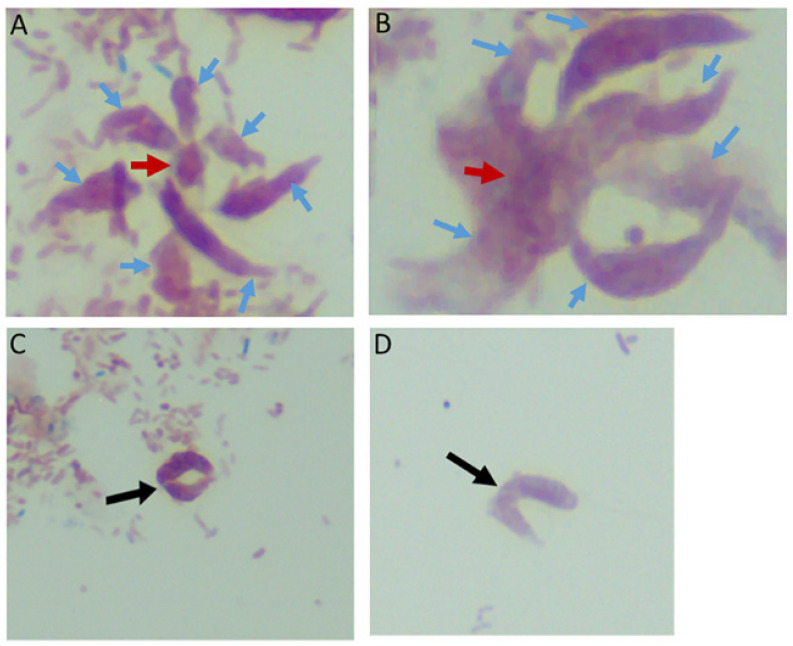
Multiple *Colpodella* sp. (ATCC 50594) trophozoites (blue arrows) feeding on a single *P. caudatus* prey (red arrowhead) in panels (**A**,**B**). Paired juveniles still attached after egress from the cyst were observed with point of attachment shown by the black arrow in panels (**C**,**D**).

Sam-Yellowe’s trichrome staining protocols were used to stain formalin-fixed cells of *Colpodella* sp. (ATCC 50594) from di-protist cultures. Two major life cycle stages occur in *Colpodella* sp. (ATCC 50594) and the staining protocol distinguishes these. The stages are trophozoites and cysts of predator and prey. Figure 1 shows cysts and trophozoites of *Colpodella* sp. (ATCC 50594), and *Colpodella* sp. (ATCC 50594) trophozoites attached to *P. caudatus* prey. Black arrows show young demilune cysts of *Colpodella* sp. (ATCC 50594) (panels A–D, G). *Colpodella* sp. (ATCC 50594) cysts have a large clear zone surrounding the cyst, separating the cysts from bacteria. Mature cysts of *Colpodella* sp. (ATCC 50594) are identified by black arrowheads (panels B, D and G) and *P. caudatus* cysts are identified by the red arrowhead (panel A). Predator (yellow arrow)-prey (red arrow) in myzocytosis with tubular tethers of varying lengths (open black arrows), used for attachment, are shown in panels E and F. A large posterior food vacuole (Fv) is formed in the predator as cytoplasmic contents are aspirated from the prey. Multiple predators can attach to one prey at a time for feeding in the process of myzocytosis. Figure 2 shows as many as seven predators attached to one prey (Figure 2A, blue arrows, red arrowhead indicates prey) and six predators attached to one prey in Figure 2B (blue arrows, red arrow indicates prey). Egressed trophozoites from *Colpodella* sp. (ATCC 50594) cysts were identified, still attached at the anterior ends (Figure 2C,D).

### 3.2. Time Course Experiments to Determine Duration of Colpodella sp. (ATCC 50594) Life Cycle

In order to identify the timing and stage transitions within the life cycle of *Colpodella* sp. (ATCC 50594), time course experiments were performed. We sought to determine at what time point each life cycle stage occurred and to identify the predominant cyst stage during the resting stage of the life cycle. Three time course experiments were performed. In the first experiment, four replicates each lasting 36 h was performed. Cells were collected every four hours, formalin-fixed and stained with Sam-Yellowe’s trichrome to observe the development of *Colpodella* sp. (ATCC 50594) trophozoite and cyst stages (Supplementary Table S1). At zero hours after subculture a few *P. caudatus* and *Colpodella* sp. (ATCC 50594) cysts were present. From 4 to 8 h, there were predominantly *P. caudatus* trophozoites, and only few *Colpodella* sp. (ATCC 50594) trophozoites. From 8 to 20 h the number of *Colpodella* sp. (ATCC 50594) trophozoites increased along with mature *Colpodella* sp. (ATCC 50594) cysts. By 24 h most *Colpodella* sp. (ATCC 50594) trophozoites observed were in myzocytosis with *P. caudatus*, representing the most active time point of the life cycle (Table S1). Cells in myzocytosis were still observed up to 32 h, along with encystation of *Colpodella* sp. (ATCC 50594) and pre-cyst stages of *Colpodella* sp. (ATCC 50594). Young trophozoites and cysts of *Colpodella* sp. (ATCC 50594) were observed at 36 h.

### 3.3. Time Course Experiments to Determine Life Cycle Stage Differentiation of Colpodella sp. (ATCC 50594)

**Figure 3:**
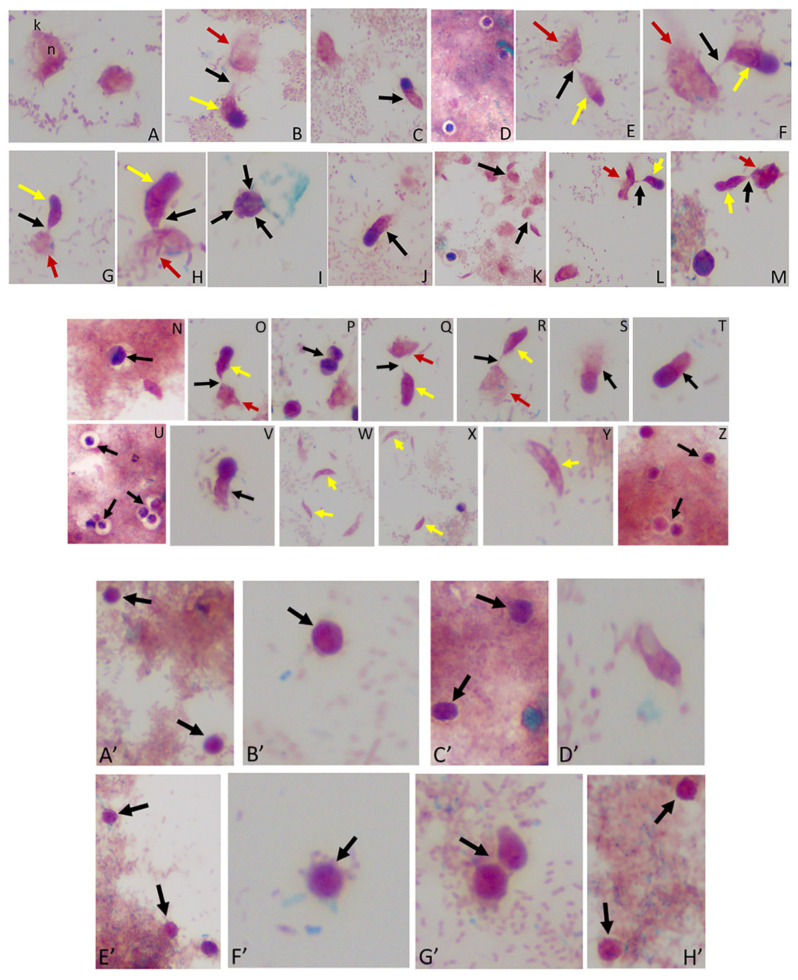
(**A**–**M**): Time course experiments showing development and timing of life cycle stages (**A**,**B** 20 h), (**C**,**D** 22h), (**E**,**F** 24 h), (**G**–**I** 25 h), (**J**,**K** 26 h), and (**L**,**M** 27 h). Kinetoplast (k) and nucleus (n) of *Parabodo caudatus* is shown in panel (**A**). Black arrows in panels (**B**,**E**–**G**,**H**,**K**–**M**) identify tubular tethers between *Colpodella* sp. (ATCC 50594) (yellow arrows) and *P. caudatus* (red arrows). Black arrowheads identify pre-cysts. A mature cyst of *Colpodella* sp. (ATCC 50594) is shown in panel I. Young demilune cysts of *Colpodella* sp. (ATCC 50594) shown in panel (**D**). (**N**–**Z**): Time course experiments showing development and timing of life cycle stage transitions in culture. (**N**,**O** 28 h), (**P**,**Q** 29 h), (**R**–**T** 30 h), (**U**,**V** 32 h), (**W**,**X** 34 h) and (**Y**,**Z** 36 h). Young demilune cysts are shown in panels (**N**,**P**,**U**). Yellow arrows identify *Colpodella* sp. (ATCC 50594) trophozoites in myzocytosis with *P. caudatus* prey (red arrows) in panels (**O**,**Q**,**R**). Black arrows identify tubular tethers. Black arrowheads in panels (**S**,**T**,**V**) identify pre-cysts of *Colpodella* sp. (ATCC 50594). Young trophozoites of *Colpodella* sp. (ATCC 50594) were identified in panels (**W**–**Y**) and mature cysts of *Colpodella* sp. (ATCC 50594) were seen in panel (**Z**). Zones of inhibition/clear zones were observed surrounding and separating cysts of *Colpodella* sp. (ATCC 50594) from bacteria (antibacterial zone). (**A’**–**H’**): Time course experiments showing development and timing of life cycle stage transitions in culture. (**A’**,**B’** 38 h), (**C’**,**D’** 40 h), (**E’**,**F’** 5 days) and (**G’**,**H’** 7 days). Mature cysts of *Colpodella* sp. (ATCC 50594) are shown in panels (**A’**–**E’**). Resting cysts of *Colpodella* sp. (ATCC 50594) are shown in panels (**F’**−**H’**).

Time course two was performed to understand life cycle stage differentiation at the most active stages of the *Colpodella* sp. (ATCC 50594) life cycle. The four replicates of time course 1 were very similar in the timing and life cycle stages represented, as shown in Table S1. Time course two lasted for 40 h and cells were formalin-fixed and stained every four hours till 22 h. Between 22 and 28 h, cells were collected and formalin-fixed every hour. After 28 h, cells were collected every two hours till 40 h. Representative life cycle stages observed, and description of stages are shown in Figure 3A–Z and Table S2. *Parabodo caudatus* trophozoites and *Colpodella* sp. (ATCC 50594) in myzocytosis were identified at 20 h (T = 5, Figure 3A,B), *Colpodella* sp. (ATCC 50594) pre-cysts and cysts were identified (panels C and D), with the active feeding by myzocytosis observed from 22 (T = 6) to 30 h (T = 13) (Figure 3E–T). Cells were found to be most active during the 20 h to 28 h time points. Multiple myzocytosis attachments were observed. Early and mature cysts were also seen as *Colpodella* sp. (ATCC 50594) encysted. Pre-cysts of *Colpodella* sp. (ATCC 50594) were observed (panels 3C and 3J). In cells undergoing myzocytosis, young and mature cysts were observed up to 30 h (T = 13). By 32 h, more *Colpodella* sp. (ATCC 50594) cysts were observed and young trophozoites excysting from cysts were observed (panels 3U to 3Y). By 36 h, cyst stages of both *Colpodella* sp. (ATCC 50594) and *P. caudatus* were present in the culture and remained predominant up to 40 h (T-18). Clear zones separating *Colpodella* sp. (ATCC 50594) cysts from bacteria were observed (panels 3D and 3U).

### 3.4. Time Course to Determine the Predominant Resting Colpodella sp. (ATCC 50594) Cyst Stage in Culture

A third time course was performed to identify the predominant *Colpodella* sp. (ATCC 50594) cyst stage in a resting culture. Cells were formalin-fixed for staining every 24 h for eight days. Additionally, cysts from day five and cysts from day seven resting cultures were each pooled and formalin-fixed for trichrome staining. The results show that the predominant *Colpodella* sp. (ATCC 50594) life cycle stage in resting cultures for up to eight days were mature cysts with a single nucleus. Cysts on days seven and eight were the same stage. A few mature cysts were observed with two or more nuclei and some young demilune cysts were also observed (Figure 3A’–H’). In order to determine the percentage of different cyst stages present in the resting culture, cysts from pooled day five and seven cultures were counted. One hundred cysts were counted on duplicate slides from cysts stained from day 5 and 7 cultures. Of the cysts, 85% were mature cysts on day 5 and 93% on day 7 (Table 1). A few *Colpodella* sp. (ATCC 50594) and *P. caudatus* trophozoites excysted during these time points.

### 3.5. Differential Interference Microscopy (DIC)

**Figure 4:**
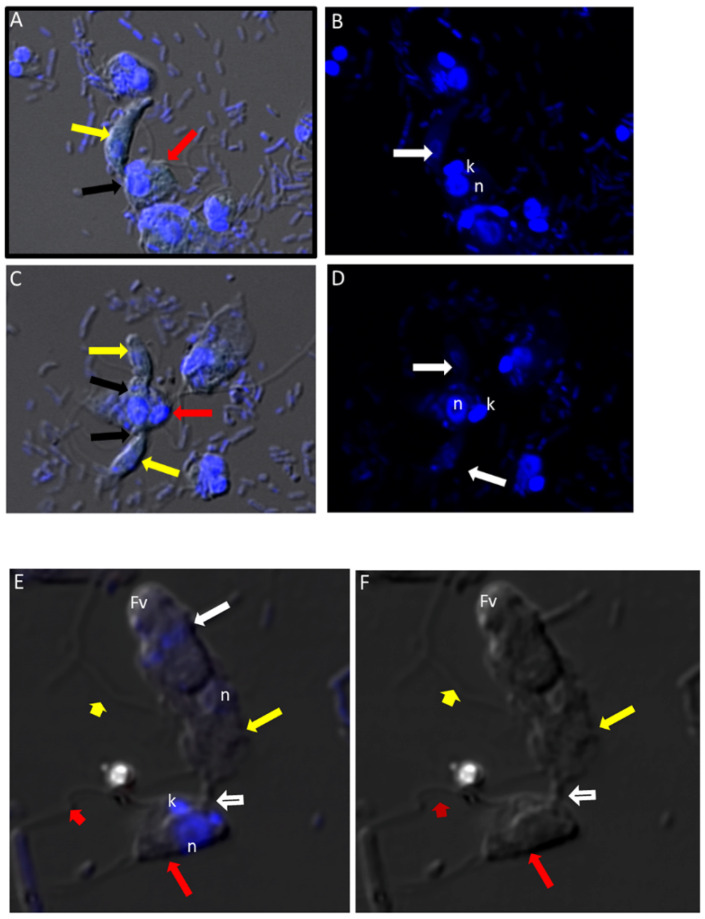
(**A**–**D**): Differential Interference contrast (DIC) microscopy of DAPI stained *Colpodella* sp. (ATCC 50594) life cycle stage transitions. (**A**,**C**) Merger of DIC and DAPI stained *Colpodella* sp. (ATCC 50594) trophozoites (yellow arrow) and *P. caudatus* prey (red arrow) in myzocytosis. Two predators feeding on one prey are shown in panel (**C**). The tubular tether joining predator and prey is indicated by the black arrow. (**B**,**D**) DAPI stained nuclei and kinetoplast. The kinetoplast (k) and nucleus (n) of *P. caudatus* is identified in panels (**B**,**D**). The central nucleus of *Colpodella* sp. (ATCC 50594) trophozoites is indicated by the white arrows. (**E**,**F**): Differential Interference contrast (DIC) microscopy of DAPI stained *Colpodella* sp. (ATCC 50594) life cycle stage transitions. (**E**) Merger of DIC and DAPI. (**F**) DIC alone. *Colpodella* sp. (ATCC 50594) trophozoite (yellow arrow) shown in myzocytosis with *P. caudatis* (red arrow). The tubular tether between predator and prey is indicated by the open arrow. DAPI stained aspirated contents can be seen at the posterior food vacuole of the *Colpodella* sp. (ATCC 50594). Yellow arrowhead identifies the flagella of *Colpodella* sp. (ATCC 50594) and the flagella of the prey are indicated by the red arrowhead. The kinetoplast (k) and nucleus of the prey and the nucleus of *Colpodella* sp. (ATCC 50594) were identified.

**Figure 5:**
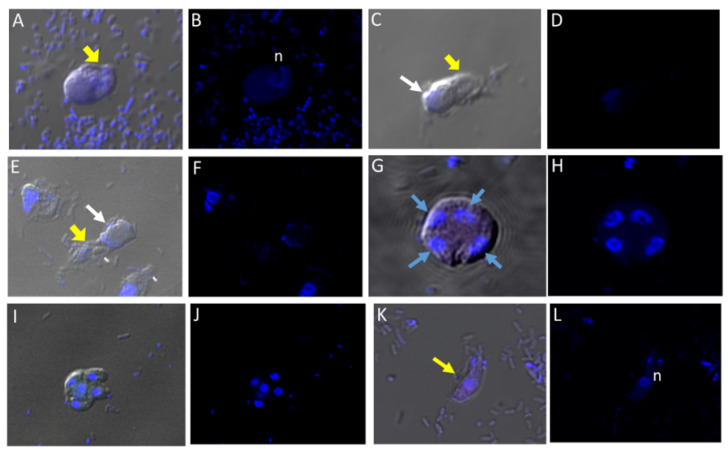
(**A**–**L**): Differential Interference contrast (DIC) microscopy of DAPI stained *Colpodella* sp. (ATCC 50594) life cycle stage transitions. DIC and DAPI staining of pre-cyst and cyst stages of *Colpodella* sp. (ATCC 50594). Yellow arrowhead shows frayed anterior end of encysting trophozoites in panels (**A**,**C**,**E**). White arrows identify the DAPI stained aspirated contents from the prey in the posterior food vacuole. A four-nuclei cyst is identified in panels (**G**,**H**), a five-nuclei cyst in panels (**I**,**J**) and a young trophozoite in panels (**K**,**L**). The central nucleus (n) was identified.

We performed DIC microscopy and DAPI staining to identify the morphology of life cycle stages and the number of nuclei present in trophozoites and cysts, respectively. Single or double *Colpodella* sp. (ATCC 50594) (yellow arrows) feeding on *P. caudatus* (red arrow) were identified. DAPI staining identified the kinetoplast and nucleus of *P. caudatus* and the central nucleus of *Colpodella* sp. (ATCC 50594) trophozoites (white arrow). DIC microscopy and DAPI staining are shown in Figure 4A,C and DAPI staining alone shown in panels Figure 4B,D. A *Colpodella* sp. (ATCC 50594) trophozoite (yellow arrow) feeding on *P. caudatus* (red arrow) in myzocytosis is shown by DIC and DAPI staining in Figure 4E. The posterior food vacuole (Fv) in *Colpodella* sp. (ATCC 50594) is prominent. The tubular tether joining predator and prey was identified (open white arrow). DAPI stained aspirated cytoplasmic contents from the prey are shown in *Colpodella* sp. (ATCC 50594) by the grey arrow. Yellow arrowheads identify *Colpodella* sp. (ATCC 50594) flagella and red arrowheads identify *P. caudatus* flagella. The tubular tethers are flexible and of varying lengths (Figure S1). DIC microscopy and DAPI stained pre-cysts of *Colpodella* sp. (ATCC 50594) are shown in Figure 5A,C,E. The yellow arrow shows the frayed and disintegrated anterior end of the trophozoite and the forming cyst. Panels B, D and F show DAPI stained nucleus and aspirated cytoplasmic contents of the prey, respectively. DIC microscopy and DAPI staining show a four-nuclei cyst (Figure 5G) and a five-nuclei cyst (Figure 5I). The nuclei in each cyst were identified by the blue DAPI staining. A young trophozoite identified by DIC microscopy and DAPI staining shows a central nucleus (Figure 5K,L).

### 3.6. Transmission Electron Microscopy

**Figure 6:**
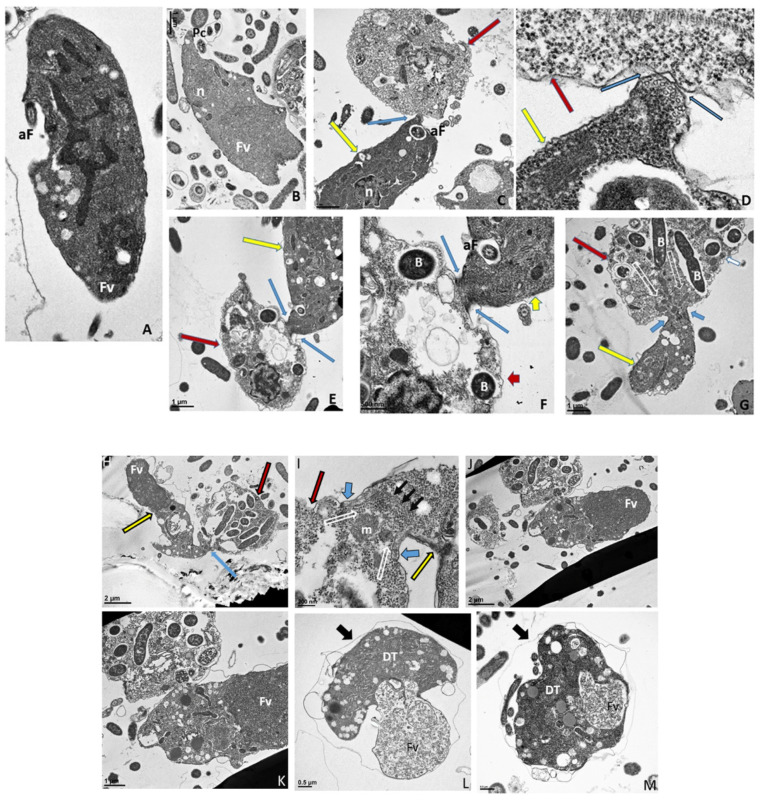

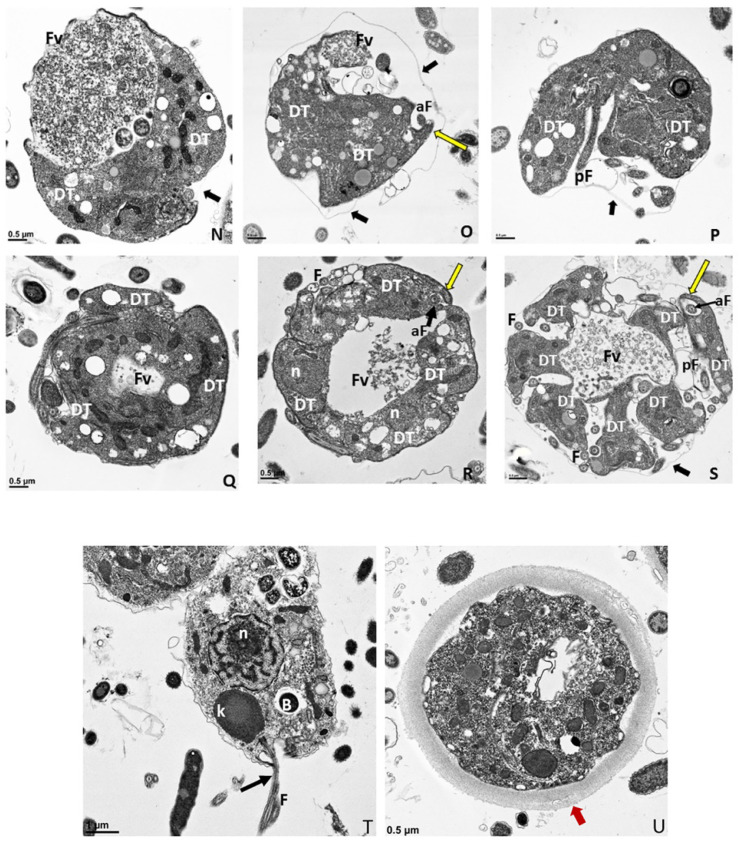
(**A**–**G**): Transmission electron micrographs of *Colpodella* sp. (ATCC 50594) life cycle stages. (**A**,**B**), Trophozoites. (**C**), attachment (**D**, enlarged) of *Colpodella* sp. (ATCC 50594) trophozoite (red arrow) to *P. caudatus* showing two points of attachment to the plasma membrane of *P. caudatus* (yellow arrow). Microtubules and organelles are shown at the point of attachment. (**E**), (enlarged **F**) microtubules spread out at point of attachment. (**G**), opening shown with cytoplasmic contents of prey aspirated into *Colpodella* sp. (ATCC 50594) predator. Open arrows show direction of flow of cytoplasmic contents. Bacteria (**B**) in prey were identified. Blue arrows shown points of attachment in predator-prey pairs undergoing myzocytosis. Anterior flagella (aF), developing trophozoite (DT), flagella (F), food vacuole (Fv), nucleus (n), pseudo-conoid (PC), posterior flagella (pF). Scale bars, (**A**) (2 µm), (**B**) (1 µm), (**C**) (2 µm), (**D**) (500 nm), (**E**) (1 µm), (**F**) (500 nm), and (**G**) (1 µm). (**H**–**M**): Transmission electron micrographs of *Colpodella* sp. (ATCC 50594) life cycle stages. (**H**) (enlarged in **I**) shows attachment of *Colpodella* sp. (ATCC 50594) trophozoites (yellow arrow) to *P. caudatus* (red arrow) containing rod shaped bacteria in the cytoplasm. Blue arrow shows point of attachment between predator and prey. The plasma membrane of *Colpodella* sp. (ATCC 50594) is seen surrounding the plasma membrane of *P. caudatus* (three small black arrows) being pulled into the cytoplasm of *Colpodella* sp. (ATCC 50594). Open white arrows show the direction of flow of the cytoplasmic contents from the prey. (**J**), (enlarged in **K**) shows the posterior food vacuole of *Colpodella* sp. (ATCC 50594) enlarged after feeding accompanied by disintegration of the anterior end with loss of flagella and apical organelles. (**L**), young cyst of *Colpodella* sp. (ATCC 50594) with developing trophozoite (DT) and remnant food vacuole. M, young cyst with developing trophozoite and remnant food vacuole. Anterior flagella (aF), developing trophozoite (DT), flagella (F), food vacuole (Fv), mitochondria (m), nucleus (n), pseudo-conoid (PC), posterior flagella (pF). Scale bars, (H) (2 µm), (**I**) (200 nm), (**J**) (2 µm), (**K**) (1 µm), (**L**,**M**) (0.5 µm). (**N**–**S**): Transmission electron micrographs of *Colpodella* sp. (ATCC 50594) life cycle stages. N-P, shows *Colpodella* sp. (ATCC 50594) mature cysts containing two developing trophozoites with remnant food vacuoles (**N**,**O**), black arrows identify the thin cyst wall and the developing pseudo-conoid (yellow arrow) was identified in a developing trophozoite. (**Q**–**S**) show different number of developing trophozoites in cysts of *Colpodella* sp. (ATCC 50594). (**Q**), cyst with three DT, (**R**), cyst with four DT and (**S**), cyst with seven DT. One trophozoite is more mature than the other six, and contains a developed pseudo-conoid (yellow arrow), in an asymmetric and asynchronous development of the developing trophozoites. Remnant food vacuoles were identified in panels Q-S. Black arrows identify the thin cyst wall of *Colpodella* sp. (ATCC 50594) cysts. Anterior flagella (aF), developing trophozoite (DT), flagella (F), food vacuole (Fv), mitochondria (m), nucleus (n), pseudo-conoid (PC), posterior flagella (pF). Scale bars, N to S (0.5 µm). (**T**–**U**): (**T**), Trophozoite of *P. caudatus* showing the nucleus (n), kinetoplast (k), flagella (F) and black arrow is paraflagellar rod. (**U**), Cyst of *P. caudatus* showing the thick cyst wall (red arrowhead). Bacteria (B) were identified in the cytoplasm of *P. caudatus.* Scale bars, (**T**) (1 µm) and (**U**) (0.5 µm).

Cells from di-protist cultures were prepared for TEM to investigate the ultrastructure of *Colpodella* sp. (ATCC 50594) life cycle stages. *Colpodella* sp. (ATCC 50594) trophozoites were identified in Figure 6A,B (Figure S6A). Organelles indicated by black arrows are rhoptries with the bodies extending into the cytoplasm. *Colpodella* sp. (ATCC 50594) trophozoites (yellow arrows) and *P. caudatus* (red arrows) in myzocytosis were also identified in Figure 6C (enlarged in Figure 6D and Figure S6B), in Figure 6E (enlarged in Figure 6F) and in Figure 6G. Initial contact is made by the pseudo-conoid of *Colpodella* sp. (ATCC 50594) to the plasma membrane of *P. caudatus*. The point of attachment is indicated by blue arrows. Microtubular organization in the cytoskeleton in areas of close proximity to the point of attachment is seen in panels 6 D to G (Figures S6B and S7A,B). The flow of cytoplasmic contents from the prey (open white arrows) into the predator was identified in Figure 6G (enlarged in Figure S7B). The flow of cytoplasmic contents from the prey into *Colpodella* sp. (ATCC 50594) (white arrow) was observed (Figure S6C). The plasma membrane of the prey pulled into the predator is indicated by black arrows. The initial attachment of the pseudo-conoid with bands of microtubules organized at the point of attachment (Figure S7A) and extension of the plasma membrane of *Colpodella* sp. (ATCC 50594) (blue arrows) with foci of microtubules is shown (Figures S6C and S7B) in a two-step process for myzocytosis. The plasma membrane of the prey pulled into the cytoplasm of the predator is broken down to allow for aspiration of cytoplasmic contents of the prey (Figure S7B). Bacteria (B) taken up by *P. caudatus* were observed in the cytoplasm.

Formation of the large posterior food vacuole (Fv) in *Colpodella* sp. (ATCC 50594) (yellow arrow) is seen in Figure 6H. A high magnification of the tubular tether holding predator and prey together shows the plasma membrane and cytoplasm of *P. caudatus* being pulled into the predator surrounded by the plasma membrane of the predator (Figure 6I, large black arrowheads). The direction of flow of cytoplasmic contents, including mitochondria (m) and other organelles, aspirated from the prey into the cytoplasm of the prey is shown (blue arrows, Figure 6I, enlarged in Figure S6C) and Figure S7B.

Following myzocytosis, the anterior end of the *Colpodella* sp. (ATCC 50594) trophozoite disintegrates resulting in loss of the flagella and organelles to form the pre-cyst formed from the posterior food vacuole (Fv), remnant cytoplasm and nucleus (Figure 6J and enlarged in Figure 6K). The young cyst stages of *Colpodella* sp. (ATCC 50594) with developing trophozoites (DT) are shown in Figure 6L,M with the residual food vacuole (Fv) and a thin cyst wall (black arrow). Cysts containing two developing trophozoites (Figure 6N–P), three (Figure 6Q), four (Figure 6R) and seven (Figure 6S) developing trophozoites are shown. *Colpodella* sp. (ATCC 50594) cysts can have both asymmetric and symmetric division as both odd and even-numbered juvenile trophozoites were observed within mature cysts. In Figure 6O,P,R,S, juvenile trophozoites at different stages of maturity were observed. Asynchronous development was observed where one trophozoite already had a developed pseudo-conoid. The developed pseudo-conoid in the cysts indicated by yellow arrows was identified along with flagella (anterior and posterior).

The thin cyst wall is indicated by black arrows. A trophozoite of *P. caudatus* is shown in Figure 6T. The kinetoplast (k), nucleus (n), bacteria (B) in the cytoplasm and flagellum (F) are shown. The thick cyst wall of *P. caudatus* (Figure 6U) can be differentiated from the thin cyst wall of *Colpodella* sp. (ATCC 50594).

### 3.7. Immunofluorescence of Colpodella sp. (ATCC 50594) in Diprotist Culture Using Anti-RhopH3 Antibodies

In order to determine if proteins associated with host cell invasion in pathogenic apicomplexans are shared by the free-living *Colpodella* sp. (ATCC 50594), we performed immunofluorescence assay (IFA) using *Plasmodium* sp. and *Toxoplasma gondii* specific antibodies (Table 2). We investigated cross-reactivity of the antibodies to proteins in different life cycle stages of *Colpodella* sp. (ATCC 50594). 

**Figure 7:**
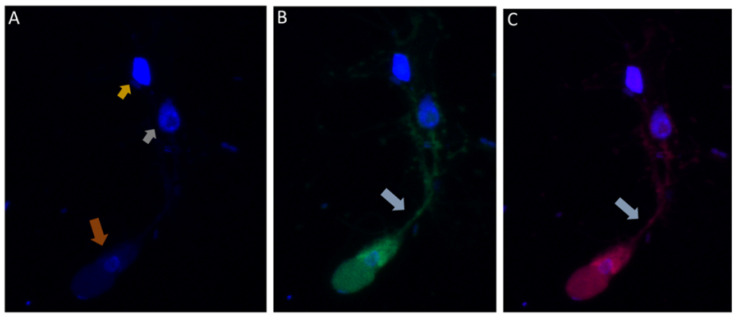

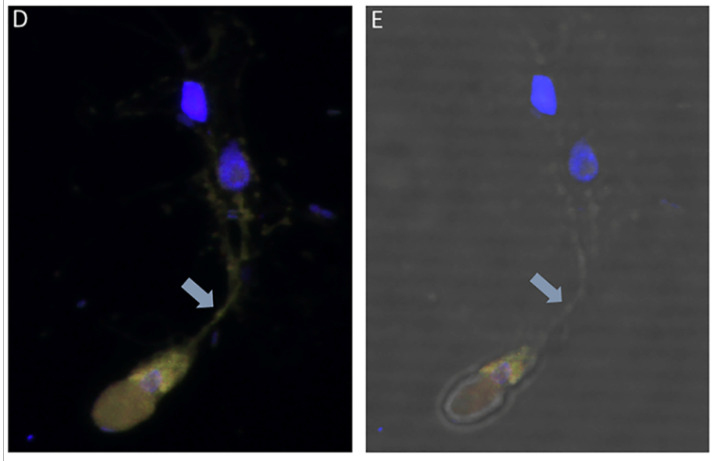
(**A**–**E**): Immunofluorescence microscopy of *Colpodella* sp. (ATCC 50594) trophozoite in myzocytosis using antiserum 686 (1:100 dilution) specific for *P. falciparum* rhoptry protein, RhopH3 (green) and antiserum FL (1:100 dilution) specific for *P. berghei* RhopH3 (red). DAPI and DIC images are also shown. (**A**). DAPI staining showing nucleus of *Colpodella* sp. trophozoite (red arrow), nucleus (grey arrowhead) and kinetoplast (gold arrowhead) of *P. caudatus* trophozoite. (**B**). Antiserum 686 cross reacted with *Colpodella* sp. (ATCC 50594) proteins in the cytoplasm and in the tubular tether used for attachment with the prey. (**C**). Antiserum FL cross reacted with *Colpodella* sp. (ATCC 50594) proteins in the cytoplasm and in the tubular tether used for attachment with the prey and is colocalized with antiserum 686. (**D**). merge of DAPI, 686 and FL showing antibody colocalization of antibody reactivity. (**E**). merge of DAPI, 686, FL and DIC showing antibody reactivity and morphology of *Colpodella* sp. (ATCC 50594) and *P. caudatus* trophozoites in myzocytosis. Antibody reactivity is seen on the tubular tether and within the *P. caudatus* prey in the section closest to the predator (movie in Supplementary Figure S2).

DAPI staining identified the round central nucleus of *Colpodella* sp. (ATCC 50594) trophozoites (red arrow) and the kinetoplast (gold arrowhead) and nucleus (grey arrowhead) of prey *P. caudatus* (Figure 7A). Antiserum 686 against the RhopH3 rhoptry protein of *P. falciparum* (green) and antiserum FL against the RhopH3 protein of *P. berghei* (red) reacted with structures in the cytoplasm of *Colpodella* sp. (ATCC 50594) trophozoites. Antibody reactivity was also observed in the tubular tether with what appears to be a spherical structure in the tube. Anti-RhopH3 antibody reactivity was also observed in the cytoplasm of the prey (Figure 7B,C). Antibody/DAPI and Antibody/DAPI/ DIC merged images identified colocalization of the two RhopH3 specific antibodies, the morphology of the predator and prey, the long tubular tether and the enlarged posterior food vacuole of the predator (Figure 7D,E). Volocity videos generated from z-stack acquisitions of immunofluorescence, DAPI and DIC images show the predator and prey in myzocytosis (Figure S2). Antibody 686 and 676 reactive with *P. falciparum* RhopH3 and rhoptries, respectively, reacted with discrete structures at the apical end of the attached *Colpodella* sp. (ATCC 50594) trophozoites and within the cytoplasm of the trophozoite (Figures S3 and S4). Open arrows show the point of attachment (Figure S3). Volocity videos generated from z-stack acquisitions of immunofluorescence, DAPI and DIC images show the predator and prey in myzocytosis (Figure S4). Still images of Figure S4 are shown in Figure S5 and identify the prey’s destruction upon aspiration by the predator. Cytoplasmic contents of *Colpodella* sp. (ATCC 50594) identified by antibody reactivity as particulate circular structures could be seen (Figures S4 and S5).

### 3.8. Immunofluorescence of Colpodella sp. (ATCC 50594) with Antibodies against IMC3, IMC7, Py235, EBA175, AMA1 and Plasmepsin II Proteins

**Figure 8:**
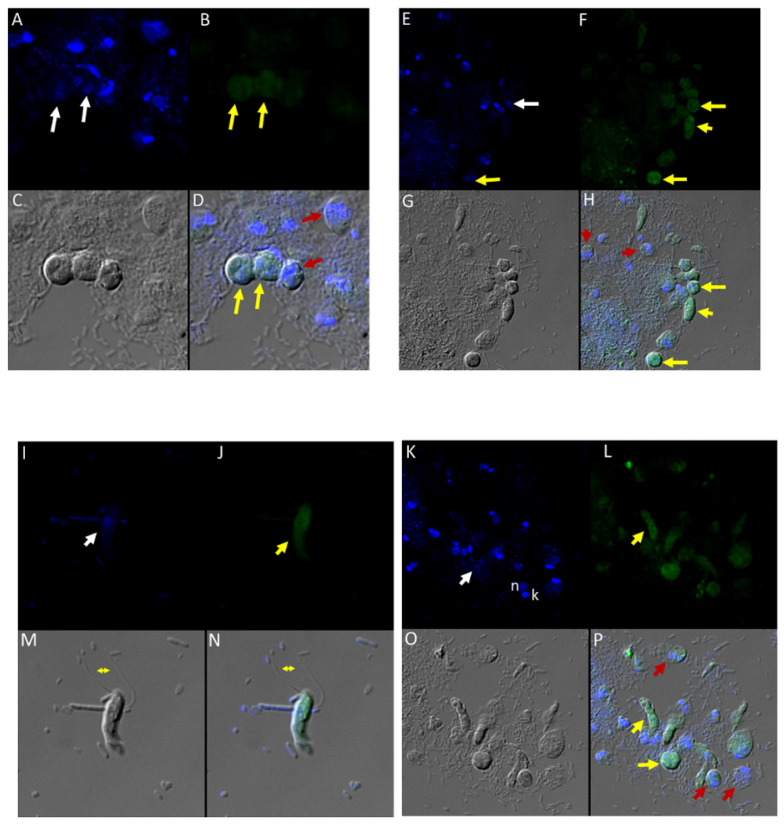
(**A**–**H**): Immunofluorescence microscopy of *Colpodella* sp. (ATCC 50594) trophozoites and cysts using anti-IMC3 antibody. (**A**,**E**); two-nuclei cysts of *Colpodella* sp. (ATCC 50594) were identified by DAPI staining (white arrows, panel **A**), single nucleus cyst is shown in panel (**E**) (white arrow). (**B**,**F**); Antibody reactivity was observed on cysts (yellow arrows) and trophozoites (yellow arrowheads) of *Colpodella* sp. (ATCC 50594). (**C**,**G**); DIC microscopy showing the morphology or trophozoites and cysts of predator and prey. (**D**,**H**); Merged antibody, DAPI and DIC images showing no reactivity of anti-IMC3 antibodies with *P. caudatus* cysts (red arrows, panel **D**) and trophozoites (red arrowheads, panel **H**), antibody reactivity with *Colpodella* sp. (ATCC 50594) cysts (panels **D** and **H**, yellow arrows). (**I**–**P**): Immunofluorescence microscopy of *Colpodella* sp. (ATCC 50594) trophozoites and cysts using anti-IMC3 FLR antibody. (**I**,**K**); DAPI stained nuclei of *Colpodella* sp. (ATCC 50594) trophozoites (white arrows, panels **I** and **K**) and *P. caudatus* trophozoites showing nucleus (n) and kinetoplast (k) (panel **K**). (**J**,**L**); anti-IMC3 FLR antibody reactivity is shown on trophozoites and cysts of *Colpodella* sp. (ATCC 50594) (yellow arrowheads and arrow). (**N**,**P**); Merging of antibody, DAPI and DIC image. Antibody reactivity was not observed on *P. caudatus* trophozoites (red arrowheads, panel **P**) while ant-IMC3 FLR reactivity was observed on trophozoites and cysts of *Colpodella* sp. (ATCC 50594) (yellow arrow and arrowhead). Flagella of *Colpodella* sp. (ATCC 50594) were identified (double arrow, panels **M** and **N**).

**Figure 9:**
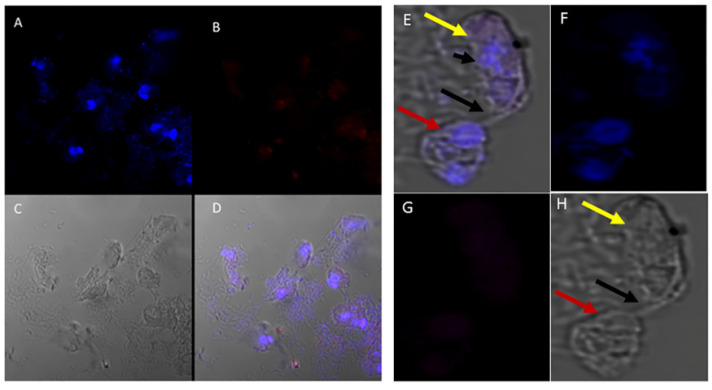
(**A**–**H**): Immunofluorescence microscopy of *Colpodella* sp. (ATCC 50594) trophozoites and cysts using anti-IMC7 and anti-Py 235 antibodies. (**A**), DAPI stained nuclei of *Colpodella* sp. (ATCC 50594) and *P. caudatus*, (**B**), Background to no reactivity was observed with anti-IMC7. Panels (**C**,**D**) shown DIC microscopy and merged Antibody, DAPI and DIC images, respectively. (**E**), Merged anti-Py235 antibody, DAPI, DIC image of *Colpodella* sp. (ATCC 50594) trophozoite (yellow arrow) and *P. caudatus* (red arrow) in myzocytosis. The tubular tether (black arrow) joining predator and prey is shown and DAPI stained aspirated cytoplasmic contents from the prey is shown (black arrowhead). (**G**), no antibody reactivity was observed and (**H**), DIC microscopy of *Colpodella* sp. (ATCC 50594) trophozoite and *P. caudatus*.

**Figure 10:**
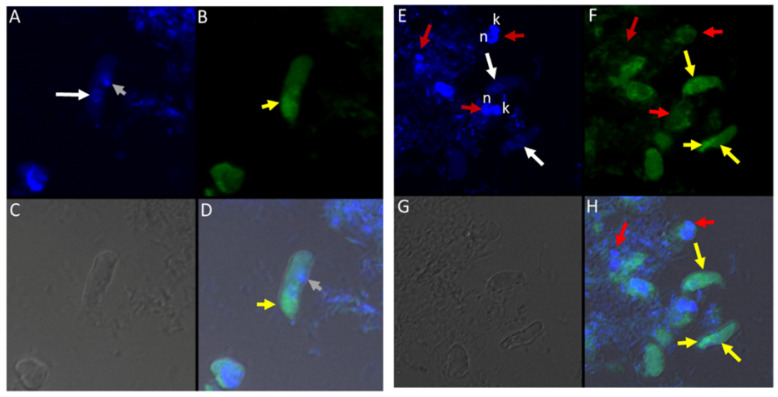
(**A**–**H**): Immunofluorescence microscopy of *Colpodella* sp. (ATCC 50594) trophozoites and cysts using anti-AMA-1antibodies. (**A**,**E**); DAPI stained nuclei of *Colpodella* sp. (ATCC 50594) (white arrow) and *P. caudatus* (red arrow) showing DAPI stained nucleus (n) and kinetoplast (k). (**B**,**F**) shows antibody reactivity with *Colpodella* sp. (ATCC 50594) trophozoites (yellow arrow) with intense antibody reactivity at the anterior end of the trophozoites (yellow arrowhead), panels (**B**,**D**,**F**,**H**). No antibody reactivity was observed on *P. caudatus* trophozoites (red arrows). (**C**,**G**); DIC microscopy shown the morphology of *Colpodella* sp. (ATCC 50594) and *P. caudatus*. (**D**,**H**); shows merged images of Antibody, DAPI and DIC. Grey arrowhead identified DAPI stained aspirated cytoplasmic contents of *P. caudatus*.

Different antibodies against known apical and non-apical proteins of *P. falciparum* and *Toxoplasma gondii* were used in IFA. We wanted to know if antigens recognized by these antibodies could be localized in *Colpodella* sp. (ATCC 50594). Antisera were diluted at 1:50, 1:100, 1:200, 1:500, and 1:1000. Anti-IMC3 FLR which recognizes the full-length antigen was reactive with proteins in the cysts and trophozoites of *Colpodella* sp. (Figure 8A–P) and weakly in *P. caudatus*. *Colpodella* sp. (ATCC 50594) two-way cysts were reactive with anti-IMC3FLR (yellow arrows). Red arrows show weak to no reactivity in *P. caudatus*. *Colpodella* sp. (ATCC 50594) trophozoites were reactive with the antibody (yellow arrowhead, Figure 8H,P)). No reactivity was obtained with *P. caudatus* trophozoites (red arrowheads, Figure 8H,P). A young *Colpodella* sp. (ATCC 50594) trophozoite reacted with anti-IMC3 FLR. Both flagella are shown (double yellow arrow, Figure 8M,N). IMC3 is an inner membrane complex protein found in apicomplexans and was identified in *Colpodella* sp. (ATCC 50594). IMC7 is another inner membrane complex protein identified in apicomplexan parasites. There was no reactivity with anti-IMC7 antibody as the reactivity was observed as background reactivity (Figure 9A–D). There was no cross reactivity observed with Py235 antisera with proteins of *Colpodella* sp. (ATCC 50594) (Figure 9E–H). The antibody is specific for the 235 kDa rhoptry protein of *P. yoelii*, which is a rodent parasite. Only faint background reactivity was observed. The black arrow identifies the tubular tether formed between predator (yellow arrow) and prey (red arrow) Figure 9E,H. DAPI stained cytoplasmic contents aspirated from the prey were identified (black arrowhead, Figure 9E). AMA1 is a microneme protein used in cell invasion among parasitic apicomplexans. Intense cross reactivity was obtained with the antibodies on proteins of *Colpodella* sp. trophozoites and weakly on *P. caudatus* trophozoites (Figure 10A–H).

**Figure 11:**
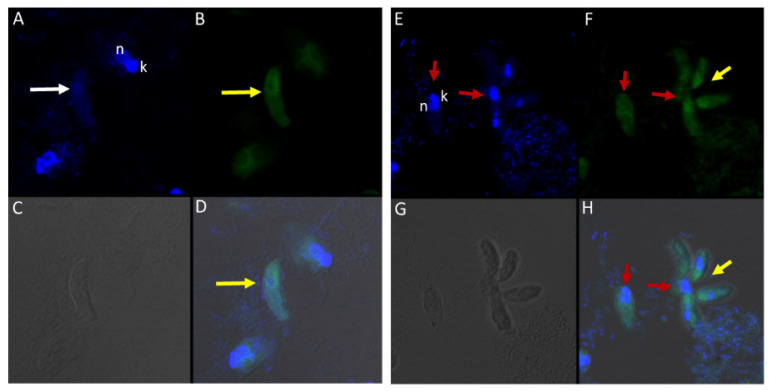
(**A**–**H**): Immunofluorescence microscopy of *Colpodella* sp. (ATCC 50594) trophozoites and cysts using anti-EBA175 antibodies. (**A**,**E**); DAPI stained nuclei of *Colpodella* sp. (ATCC 50594) (white arrow) and *P. caudatus* trophozoites (red arrow) showing nucleus (n) and kinetoplast (k). (**B**,**F**); anti-EBA175 reactivity with single *Colpodella* sp. trophozoite and multiple trophozoites feeding on a single *P. caudatus*. Yellow arrowhead shows cluster of four *Colpodella* sp. (ATCC 50594) trophozoites. (**C**,**G**); show DIC microscopy of *Colpodella* sp. (ATCC 50594) and *P. caudatus* trophozoites. (**D**,**H**); Merged image of anti-EBA175, DAPI and DIC.

**Figure 12:**
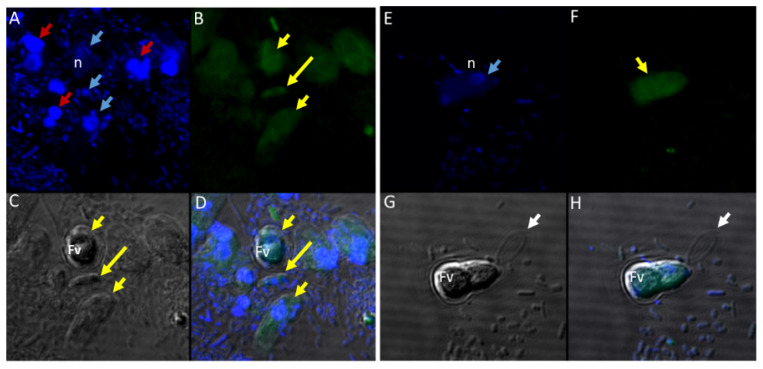
(**A**–**H**): Immunofluorescence microscopy of *Colpodella* sp. (ATCC 50594) trophozoites and cysts using anti-Plasmepsin II antibodies. (**A**,**E**); DAPI stained nuclei of *Colpodella* sp. (ATCC 50594) pre-cysts (blue arrowheads) and *P. caudatus* trophozoites (red arrowheads). (**B**,**F**); anti-plasmepsin II antibody reactivity was observed with pre-cysts of *Colpodella* sp. (ATCC 50594) with enlarged food vacuoles (Fv) after myzocytosis. Reactivity was seen in the cytoplasm at the anterior end and (**F**,**C**,**G**); DIC microscopy showing morphology of *Colpodella* sp. (ATCC 50594) and *P. caudatus*. (**D**,**H**); Merged image of anti-plasmepsin II, DAPI and DIC microscopy. Yellow arrows identified a young trophozoite that reacted with anti-plasmepsin II. Some background antibody reactivity was observed on some *P. caudatus* trophozoites.

The cross reactivity with anti-AMA1 antibody in *Colpodella* sp. (ATCC 50594) trophozoites was observed towards the apical end (Figure 10B,D,F,H, yellow arrowhead). The central nucleus of *Colpodella* sp. (ATCC 50594) trophozoites was identified by white arrows and the trophozoites showing strong anti-AMA1 reactivity are identified by yellow arrows. *Parabodo caudatus* trophozoites (red arrows) showed weak to no reactivity with the antibody. DAPI stained cytoplasmic contents aspirated from the prey were identified (Figure 10A,D, grey arrowhead). EBA175 is a microneme protein that functions in *P. falciparum* merozoite invasion. It is used to initiate invasion of red blood cells. Antibody cross reactivity was identified in *Colpodella* sp. (ATCC 50594) at the apical end of the cell and weakly in *P. caudatus* (Figure 11A–H). The DAPI stained nucleus (n) and kinetoplast (k) of *P. caudatus* and the nucleus of *Colpodella* sp. (ATCC 50594) trophozoite (white arrow) is shown in Figure 11A. Anti-EBA175 reactivity was observed in the anterior of *Colpodella* sp. (ATCC 50594) trophozoites (Figure 11B,D, yellow arrow). *Parabodo caudatus* with weak reactivity was identified (red arrows) and a group of four *Colpodella* sp. (ATCC 50594) (indicated by yellow arrowhead) feeding on a single prey (red arrow) were identified. Antibody reactivity was strongest at the apical ends of *Colpodella* sp. (ATCC 50594) trophozoites (Figure 11F,H). Plasmepsin II is an aspartic protease located in the food vacuole of *P. falciparum* where it degrades hemoglobin. Cross reactivity was observed between the antisera and antigen in *Colpodella* sp. (ATCC 50594) pre-cyst stages (Figure 12A–H, grey arrowheads). The posterior food vacuole (Fv) in the pre-cyst stages of *Colpodella* sp. (ATCC 50594) reacted with anti-plasmepsin II (Figure 12D,H). Weak to no antibody reactivity was observed in unattached *P. caudatus* trophozoites (Figure 12A, red arrowheads). The nucleus (n) of the pre-cyst was identified (Figure 12A,F) and flagella (yellow arrowhead) still present in the pre-cyst (Figure 12G,H) were observed.

**Figure 13:**
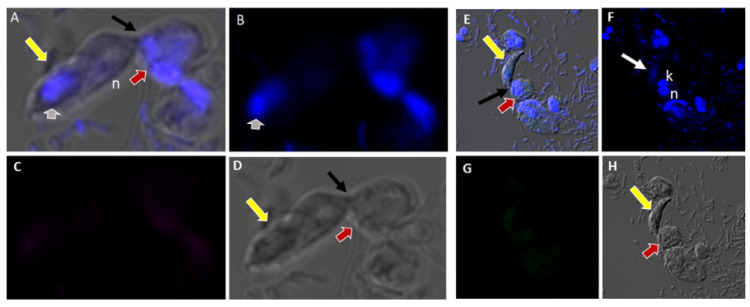
(**A**–**H**): Normal and rabbit serum negative controls. (**A**–**D**), normal mouse serum 1:100. There was no reactivity with proteins of *Colpodella* sp. (ATCC 50594) and *P. caudatus*. (**A**), merged image showing a *Colpodella* sp. (ATCC 50594) trophozoite (yellow arrow) attached to a *P. caudatus* trophozoite (red arrow). (**B**). DAPI staining shows the nuclei in both cells. The DAPI stained cytoplasmic contents aspirated from the prey can be visualized as blue in the posterior food vacuole of *Colpodella* sp. (ATCC 50594) (grey arrowhead). (**C**). There was no reactivity with NMS. (**D**). DIC showing the morphology of the cells in myzocytosis. (**E**–**H**), Normal rabbit serum, 1:100 was used as a negative control. (**E**), merged image of antibody, DAPI and DIC showing no reactivity with proteins of *Colpodella* sp. (ATCC 50594) and *P. caudatus*. The merged image shows two separate attachments of *Colpodella* sp. (ATCC 50594) and *P. caudatus* trophozoites. (**F**), DAPI staining shows the nucleus of *Colpodella* sp. (ATCC 50594) and *P. caudatus*. (**C**). Background to no reactivity was observed on cells with NRS. (**D**). DIC images show the attachment of *Colpodella* sp. (ATCC 50594) and the tubular tethers (black arrows).

There was no reactivity observed with normal mouse (Figure 13C,G) and rabbit serum (Figure 13E–H) used as negative controls. Aspiration of DAPI stained contents (grey arrowhead, Figure 13A,B) from the prey was detected in the posterior food vacuole of a *Colpodella* sp. (ATCC 50594) trophozoite (yellow arrow) attached to *P. caudatus* prey (red arrow) (Figure 13A,E). The nucleus (n) and kinetoplast (k) of *P. caudatus* and the central nucleus of *Colpodella* sp. (ATCC 50594) trophozoite (white arrow) were identified by DAPI staining.

## 4. Discussion

In this study, *Colpodella* sp. (ATCC 50594) was grown in tissue culture flasks in Hay medium [19,20] and used in studies aimed at identifying the specific time points and stage transitions for each life cycle stage. Three time course experiments with four replicates of experiment one were performed. Cells were formalin-fixed and stained with Sam-Yellowe’s trichrome [5,18] stains to view the cells. We used the first four time course replicates to demonstrate duration and reproducibility of the life cycle stages and timing of stage transitions in *Colpodella* sp. (ATCC 50594) cultures. These four replicates revealed that *P. caudatus* excysts much earlier than the predator approximately four hours after subculturing and dominates in the culture until about 20 h when majority of *Colpodella* sp. (ATCC 50594) trophozoites begin to egress from their cysts. Young trophozoites of *Colpodella* sp. (ATCC 50594) emerge and begin myzocytosis lasting between 20 and 30 h in culture. At 28 h, *P. caudatus* trophozoites begin to encyst with *Colpodella* sp. (ATCC 50594) trophozoites beginning to encyst at 30 h. By 36 h the culture is mostly quiet with only a few predator and prey trophozoites remaining. Clear zones surrounding *Colpdella* sp. (ATCC 50594) cysts, separating them from bacteria were observed. It is unclear if these zones represent anti-bacterial activity or artifacts of fixation. Additional investigations will be required to understand the identity and significance of these structures. These type of experiments have not been performed previously for any *Colpodella* species. Recent investigations in our lab identified previously undocumented life cycle stages in the di-protist culture using Sam-Yellowe’s trichrome stains, confocal and DIC microscopy and TEM [5]. The appearance of the early cyst stage of *Colpodella* sp. (ATCC 50594) shows an irregular dual-colored, dark blue-purple and white colored cyst which we designated a demilune cyst [5,18]. The stage before the demilune cyst when the food vacuole and nucleus are still visible and the anterior end of the trophozoite is disintegrated, we have designated as the pre-cyst stage. Mature *Colpodella* sp. (ATCC 50594) cysts stained dark-red blue and contained two or more juveniles [5,18]. These designations allow for stage transitions to be identified. In the current study, timing and transitions of these newly described stages were identified.

The second and third time course experiments focused on the most active time period of the life cycle and identification of the predominant cyst stage present in resting cultures, respectively. Predator and prey remain encysted until they are sub-cultured and are viable up to 14 days. The cells must be sub-cultured by 14 days or the cysts start to deteriorate, and the cell yield is low. Smears from di-protist cultures stained from pooled day five and seven cultures were counted to determine the percentage of each cyst stage present in the resting culture. Eighty to ninety percent of the *Colpodella* sp. (ATCC 50594) cysts were single nuclei mature cysts. Not all *Colpodella* sp. (ATCC 50594) cysts were mature with a single nucleus, and some had multiple asymmetric nuclei or symmetric nuclei with two or more juvenile trophozoites. Early *Colpodella* sp. (ATCC 50594) demilune cysts were also counted but there only seemed to be a relatively small number. Even though these cultures were primarily resting, free swimming predator and prey trophozoites, encysting and excysting during these time points, were also detected. The most active part of the life cycle was between 20 and 30 h, with peak activity observed between 20 and 28 h. The cultures looked the same from 36 to 40 h. Knowledge of a general life cycle reflecting transition times in culture will aid future investigations of *Colpodella* sp. (ATCC 50594) and help in the identification of infective stages in opportunistic human infections caused by *Colpodella* species. Furthermore, *Colpodella* species in ticks that feed on animals, with potential for zoonotic infections in humans, can be identified.

Single predator prey attachments were the most common pairings observed, similar to reports in other *Colpodella* species [1,3,5]. Two to three predators on one prey can also occur. In the most active stage of the life cycle observed in the present study, five to seven predators feeding on one prey were observed. Multiple attachments and different lengths of the tubular tethers have not been reported previously. One of the reasons for so many *Colpodella* sp. attaching to one prey may be connected to the smaller number of prey available when the majority of *Colpodella* sp. (ATCC 50594) trophozoites egress from cysts. *Colpodella* sp. (ATCC 50594) trophozoites exceed the number of *P. caudatus* trophozoites because of early prey encystation. This has only been observed in culture and may be different in the natural environment and in different culture conditions as summarized in Table S3. The description of the life cycle for *C. vorax* shows one to two trophozoites feeding on *P. caudatus* resulting in cysts that contain two to four juveniles [3]. Both *C. turpis* and *C. pugnax* were shown to have conjugation in their life cycles along with the formation of cysts containing two or four juveniles [2]. In *C. unguis*, oblique-transverse fission was identified in the life cycle [2,14]. In the current study, we did not observe fission or conjugation in the life cycle of *Colpodella* sp. (ATCC 50594). Knowledge of the timing of life cycle stage transitions will also facilitate investigations of organelles by isolating them. *Colpodella* species from natural habitats and their life cycle timing and stages can be investigated.

In the current study, transmission electron microscopy was performed to investigate the ultrastructure of *Colpodella* sp. (ATCC 50594) life cycle stages. Asymmetric and asynchronous multinucleate cyst stages were identified and *Colpodella* sp. (ATCC 50594) trophozoites in myzocytosis with *P. caudatus* prey were identified. Different stages of predator-prey attachment were observed, from initial attachment of the pseudo-conoid to the prey’s plasma membrane and reorganization of microtubules and apical complex organelles at the point of attachment, after which the membrane of the prey is engulfed, pulled into the predator, degraded, and cytoplasmic contents of the prey flow into the predator’s cytoplasm toward the posterior food vacuole. The extended plasma membrane of the predator with foci of microtubular organization was observed. Myzocytosis in *Colpodella vorax* also showed microtubules at the point of attachment of predator and prey [3]. In the present study, the anterior portion of the predator was observed to disintegrate after feeding, leading to loss of the flagella and cytoplasmic organelles and the rounding of the posterior food vacuole, along with the nucleus, to form the cyst.

Electron microscopy images identified the ultrastructure of *Colpodella* sp. (ATCC 50594) and *P. caudatus* cysts. The cysts of *Colpodella* sp. (ATCC 50594) have a thin cyst wall and a remnant food vacuole associated with undifferentiated trophozoites. The electron microscopy images confirmed that cysts of *Colpodella* sp. (ATCC 50594) can have asymmetric division as shown previously [5]. Results also showed that mitosis in mature *Colpodella* sp. (ATCC 50594) cysts is asynchronous. Not all the trophozoites develop and differentiate at the same rate within the cyst. Inside the cysts *Colpodella* sp. (ATCC 50594) trophozoites that are more developed can be visualized showing a pseudo-conoid, mitochondria, and defined nucleus. *P. caudatus* cysts can be differentiated by the thickness of their cyst wall. They are also seen to have a nucleus and kinetoplast. Both protists show parts of their flagella inside the cysts.

**Figure 14:**
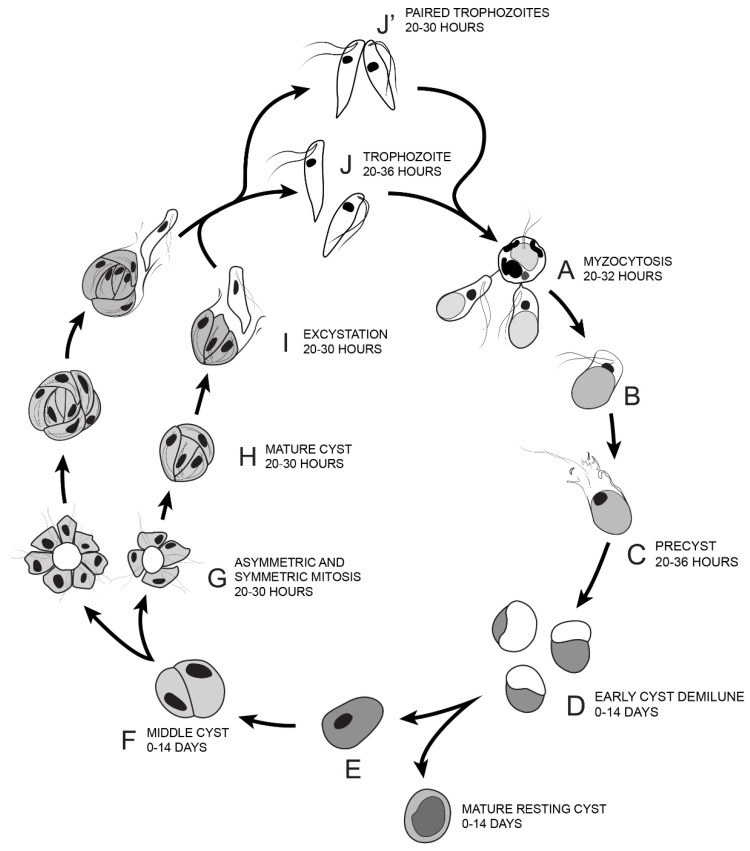
Illustration of the life cycle of *Colpodella* sp. (ATCC 50594) cultured in Hay medium. (**A**). Once trophozoites egress from cysts they begin feeding on *P. caudatus* prey. Two trophozoites are depicted feeding in the process of myzocytosis. Active cultures with predator-prey attachments are seen 20–32 h after subculture. The posterior food vacuole in *Colpodella* sp. (ATCC 50594) enlarges during feeding. (**B**). Encystment begins after feeding. (**C**). Pre-cyst showing anterior portion of trophozoite with a frayed appearance. Pre-cysts are seen 20–36 h after subculture. (**D**). Early demilune cysts can be seen in culture during the active phase of the culture or in resting cultures (0–14 days after subculture). (**E**). Mature cyst stages found in active and resting cultures can be seen in culture 0–14 days after subculture. Mature *Colpodella* sp. (ATCC 50594) cysts with two or more nuclei are observed in active cultures, as transient cysts are formed at this stage. The one nucleus cyst is predominant in the resting culture. (**F**). Two-nuclei cysts of *Colpodella* sp. (ATCC 50594) can be seen in culture, 0–14 days after subculture. (**G**). Following mitosis, development of the four-nuclei cyst is most common. Asymmetric cysts containing three, five and seven nuclei were observed in culture, in addition to symmetric cysts containing four, six or eight nuclei. Four and seven nuclei cysts are depicted and can be seen in culture 20–30 h after subculture. (**H**). Mature cysts with young trophozoites can be seen 20–30 h after subculture. (**I**). Excystation (egress) and release of young trophozoites. (**J**). Juvenile trophozoites can egress individually at 20–36 h after subculture or rarely, juvenile trophozoites in pairs still attached can egress from cysts and complete cytokinesis outside the cyst (**J’** seen 20–30 h after subculture). Paired trophozoites can egress from symmetric or asymmetric cysts. Free swimming young trophozoites egressed from cysts attach to prey to repeat the life cycle. The life cycle of *Colpodella* sp. (ATCC 50594) lasts 36 h.

Based on the life cycle stages identified by Sam-Yellowe’s trichrome staining, DIC microscopy, DAPI staining, ultrastructure investigated by TEM and time course experiments, the transitions through the life cycle of *Colpodella* sp. (ATCC 50594) are summarized as shown from steps A–J’ in Figure 14. *Colpodella* sp. (ATCC 50594) trophozoites attach to *P. caudatus* prey (A) and feed by myzocytosis, after which trophozoites begin to undergo encystation by first forming the pre-cyst (B, C) which differentiates into the early demilune cyst (D). The most active stage of the life cycle in culture is between 20–30 h after subculture with peak activity at 20–28 h. *Colpodella* sp. (ATCC 50594) encyst in culture to form transient and long-term cysts. Transient cysts excyst and release trophozoites in the active phase of the culture. Long term (mature resting) cysts can survive in culture for up to 14 days (E). The demilune cysts (D) mature and divide to form two trophozoites (F) then divide to form more than two trophozoites (G, H). Multinucleate cysts can contain up to ten nuclei. Cysts with four and seven nuclei are depicted (G, H). Trophozoites egress as individual trophozoites (I, J) or egress as a pair still attached at the anterior end with incomplete cytokinesis (J’). The duration of the life cycle in culture is 36 h.

Not much is known about the process of cell division inside the cysts of *Colpodella* species.

Closed mitosis is characteristic of apicomplexans where the nuclear envelope remains intact and the mitotic spindle is intranuclear [30,31,32,33]. Within apicomplexan life cycles, the plastid, flagella and asexual division have been the focus of investigations in order to gain insights into the origins of intracellular parasitism [30,33]. The mechanism of myzocytosis would also be instructive in the understanding of zoite invasion into host cells, since apical complex proteins are used in both processes. Genes encoding proteins for flagella and photosynthesis were lost in some apicomplexan lineages that are obligate intracellular parasites. Conversely, genes encoding secretory proteins required for host cell invasion and intracellular survival were gained [30]. In apicomplexa, asexual division results in the release of merozoites (zoites) that invade host cells. Both intracellular apicomplexan pathogens and their free-living relatives possess an apical complex with the presence of the secretory organelles, rhoptries and micronemes whose proteins initiate host cell invasion and maintain the trophozoite within the host cell [30,33]. In the current study, transmission electron microscopy showed asynchronous budding and development of *Colpodella* sp. (ATCC 50594) trophozoites within the cyst, with immature and mature juveniles present within the same cyst. Furthermore, flagella were also identified within the cyst. Mitosis in *C. vorax* was described as semi-open resulting in two and four trophozoites [3]. Cysts of *Chromera velia* also produce more than four juveniles [34], with the development of the juveniles shown to be closely associated with flagella and apical organelle complex formation [34].

In order to identify cross reactivity of antibodies specific for invasion and food vacuole proteins of *P. falciparum* in *Colpodella* sp. (ATCC 50594) life cycle stages, we performed immunofluorescence and confocal microscopy. Antibodies specific for apical and non-apical complex proteins of *P. falciparum* and non-apical complex proteins of *Toxoplasma gondii* was used in IFA with DAPI used to stain the nuclei of the predator and prey. The morphology of each protist was identified by DIC microscopy. Antiserum 686 specific for the RhopH3 rhoptry protein reacted with *Colpodella* sp. (ATCC 50594) trophozoites in free swimming trophozoites and in trophozoites attached to *P. caudatus* in myzocytosis, similar to previous reports [18]. The spherical structures reactive with rhoptry and RhopH3 specific antibodies were recognized within the cytoplasm of *Colpodella* sp. (ATCC 50594), at the points of attachment of predator and prey and within prey attached to *Colpodella* sp. (ATCC 50594). The microneme specific antibodies against AMA-1 [25] and EBA175 [24] reacted with the apical end of *Colpodella* sp. (ATCC 50594) trophozoites. The IFA data showing cross reactivity of apical complex protein specific antibodies is suggestive of a similar use of apical complex proteins for myzocytosis as reported for zoite invasion into host cells in parasitic apicomplexa. Additional investigation using *Colpodella* sp. (ATCC 50594) specific antibodies and molecular characterizations will determine if the predator–prey interactions observed use similar mechanisms to the zoite–host cell interactions recognized in pathogenic apicomplexan where intracellular host cell infections are dominant. The use of microneme and plasmepsin II specific antibodies in immunofluorescent assay studies to characterize *Colpodella* species proteins has not been performed previously. The specific structures containing the cross-reactive proteins are unknown. However, the cross reactivity of antibodies against apical complex proteins with *Colpodella* sp. (ATCC 50594) proteins suggests that events of myzocytosis may have preceded events that led to zoite internalization within host cells in intracellular parasitism. Discrete spherical and particulate structures were identified with RhopH3 specific antibodies in *Colpodella* sp. (ATCC 50594). There are no antibodies against apical complex or food vacuole proteins of *Colpodella* species. Antibodies against IMC3 cross reacted with proteins on *Colpodella* sp. (ATCC 50594) with a more diffuse staining pattern on the cells. There was no reactivity with antibodies against IMC7 and Py235. Interestingly, the antibodies against plasmepsin II, an aspartate protease found in the food vacuole of *P. falciparum,* reacted with proteins in the pre-cyst stages of *Colpodella* sp. (ATCC 50594) suggestive of a similar localization to the posterior food vacuole of *Colpodella* sp. (ATCC 50594) [23]. In a previous study anti-EBA175 was shown to react weakly with the cysts of *Colpodella* sp. (ATCC 50594) [5].

The type of genes activated and what proteins are expressed during each stage of the life cycle of *Colpodella* sp. (ATCC 50594) are unknown. Identifying genes conserved in *Colpodella* sp. (ATCC 50594) that have been identified among the parasitic apicomplexans can help with phylogenetic studies. Reactivity of antibodies against the microneme proteins, EBA175 and AMA1 used for merozoite invasion in *P. falciparum* will need to be confirmed by identifying the genes encoding these proteins in *Colpodella* sp. (ATCC 50594). The gene encoding AMA1 is highly conserved among the parasitic apicomplexans [35,36,37]. A proteomic study of the dinoflagellate *Perkinsus marinus*, identified liver stage antigen 3 and merozoite surface protein 3 of *P. falciparum* and Py235 rhoptry protein of *P. yoelii* [38]. In previous studies and in the current study, antibodies specific for rhoptry proteins and whole rhoptries of *Plasmodium* sp. cross-reacted with apical proteins in *Colpodella* sp. (ATCC 50594) and *Voromonas pontica* [17,39,40]. These results suggest that the dinoflagellates and colpodellids with synapomorphies of the apical complex containing rhoptries and micronemes may possess conserved genes encoding rhoptry and microneme proteins. Understanding the role played by the apical complex proteins may provide additional insights to help understand the origins of intracellular parasitism.

## Figures and Tables

**Table 1:** Resting cysts of *Colpodella* sp. (ATCC 50594) and trophozoites on days 5 and 7.

Day & Slide #	Early Cyst	Mature Cyst	*Colpodella* sp. Trophozoite	*Parabodo* Trophozoite	*Colpodella* sp. Precyst
Day 5 #1	19	81	0	1	2
Day 5 #2	12	88	0	0	1
Day 5 Avg.	15	85	0	1	2
Day 7 #1	6	94	1	0	0
Day 7 #2	9	91	0	2	1
Day 7 Avg.	7	93	1	1	1

**Table 2:** Antibodies used in immunofluorescence and confocal microscopy of *Colpodella* sp. (ATCC 50594).

Antibodies	Antigen Specificty	Antibody Reactivity
Antiserum 686	*Plasmodium falciparum* 110 kDa RhopH3 rhoptry protein	Apical and cytoplasmic reactivity in trophozoites
Anti-AMA-1	*P. falciparum* apical membrane antigen-1, microneme protein	Apical reactivity in trophozoites
Anti-EBA-175	*P. falciparum* erythrocyte binding antigen 175 kDa, microneme protein	Apical reactivity in trophozoites
Anti-IMC3	*Toxoplasma gondii* inner membrane complex protein 3	Diffuse reactivity on trophozoites and cysts of *Colpodella* sp. (50594)
Anti-IMC3FLR	*T. gondii* inner membrane complex protein 3	Diffuse reactivity on trophozoites and cysts of *Colpodella* sp. (ATCC 50594)
Anti-IMC7	*T. gondii* inner membrane complex protein 7	No reactivity
Anti-Plasmepsin II	*P. falciparum* plasmepsin II, food vacuole protein	Diffuse reactivity in food vacuole in pre-cyst
Anti-Py235	*P. yoelii* 235 kDa rhoptry protein	No reactivity
Anti-RhopH3 FL	*P. berghei* 110 kDa RhopH3 rhoptry protein	Apical and cytoplasmic reactivity in trophozoites

## Data Availability

There are no additional data except for what is reported in the manuscript including supplementary tables and figures.

## Supplementary Materials

The following are available online at https://www.mdpi.com/article/10.3390/tropicalmed6030127/s1, Supplementary Figure S1A–D: DAPI staining and DIC microscopy of *Colpodella* sp. (ATCC 50594) trophozoites (yellow arrow) and *P. caudatus* (red arrow) in myzocytosis showing flexibility of tubular tether (black arrow) formed between predator and prey. Flagella of *Colpodella* sp. (ATCC 50594) (yellow arrowheads) and *P. caudatus* (red arrowheads) were identified. Actin green 488 (green) staining is seen on prey (panel B) and predator and prey (panel C). Supplementary Figure S2: Volocity movie of young trophozoite of *Colpodella* sp. (ATCC 50594) and *P. caudatus* in myzocytosis. Rabbit antiserum 686 (green) and mouse antiserum FL (red) specific for the RhopH3 rhoptry protein of *P. falciparum* and *P. berghei*, respectively, reacted with *Colpodella* sp. (ATCC 50594) proteins in the cytoplasm of young trophozoite, in the tubular tether and in the cytoplasm of the prey in the area closest to the tether. The nuclei of the predator and prey and the kinetoplast of the prey is stained by DAPI. The morphology of both cells is shown by DIC microscopy. Supplementary Figure S3: Immunofluorescence microscopy of *Colpodella* sp. (ATCC 50594) trophozoites and cysts using anti-RhopH3 antibodies. Rabbit antiserum 676 [35] (red) specific for *Plasmodium falciparum* rhoptries protein reacted with apical structures in *Colpodella* sp. (ATCC 50594) and within cytoplasm of *P. caudatus* in myzocytosis. Actin green 488 (green) staining is seen diffusely on predator and prey (panel E). Supplementary Figure S4: Immunofluorescence microscopy of *Colpodella* sp. (ATCC 50594) trophozoites and cysts using anti-RhopH3 antibodies. Rabbit antiserum 686 specific for P. falciparum RhopH3 rhoptry protein reacted with cytoplasmic structures within the body and apical end of *Colpodella* sp. (ATCC 50594) trophozoites attached to *P. caudatus* prey in myzocytosis. A, merged images of antiserum 686, DAPI and DIC. B, Antiserum 686 and DIC microscopy. C, antiserum 686. D, DIC microscopy. Volocity video of Z-stack image collection shown in Figure S5. Supplementary Figure S5: Volocity movie of *Colpodella* sp. (ATCC 50594) and *P. caudatus* in myzocytosis. Rabbit antiserum 686 specific for *P. falciparum* RhopH3 rhoptry protein reacted with cytoplasmic structures within the body and apical end of *Colpodella* sp. (ATCC 50594) trophozoites attached to *P. caudatus* prey in myzocytosis. Supplementary Figure S6: Transmission electron micrographs of *Colpodella* sp. life cycle stages. A, B *Colpodella* sp. (ATcc 50594) trophozoite showing apical organelles (black arrows), C, Enlarged image of *Colpodella* sp.(ATCC 50594) (yellow arrow) and *P. caudatus* (red arrow) in myzocytosis. Blue arrows show point of attachment, showing plasma membrane of the predator surrounding the aspirated cytoplasm and plasma membrane (black arrows) of *P. caudatus* prey moving into the cytoplasm of *Colpodella* sp. (ATCC 50594). White arrow shows direction of flow of prey’s cytoplasmic contents. Mitochondria (m) was identified being aspirated. Small white arrows identify microtubular organization at the tubular tether. Supplementary Figure S7: Transmission electron micrographs of *Colpodella* sp. (ATCC 50594) life cycle stages. A, B enlarged images of *Colpodella* sp. (ATCC 50594) (yellow arrows) and *P. caudatus* (red arrows) in myzocytosis. Bands of microtubules can be seen at the point of attachment (panel A) and plasma membrane (blue arrows) of *Colpodella* sp. (ATCC 50594) trophozoite can be seen surrounding prey. Grey arrows show direction of flow of prey’s cytoplasmic contents into the cytoplasm of *Colpodella* sp. (ATCC 50594). Bacteria (B) within the cytoplasm of *P. caudatus* were identified. Supplementary Table S1: Summary of time course experiments 1-4 to determine the duration of *Colpodella* sp. (ATCC 50594) life cycle. Supplementary Table S2. Time Course two observations during the most active parts of the *Colpodella* sp. (ATCC 50594) life cycle. Time point numbers correspond to hours/days cells were collected for staining. Table S3. Summary of isolation and culture conditions for colpodellids and chromerids.
