# Supplementary material for: Colpodella sp. (ATCC 50594) Life Cycle: Myzocytosis and Possible Links to the Origin of Intracellular Parasitism
Source: Trop Med Infect Dis. 2021 Jul 11;6(3):127. doi: 10.3390/tropicalmed6030127 (PMC8293349; doi:10.3390/tropicalmed6030127)
Supplement: Supplementary file 1 [file tropicalmed-06-00127-s001.zip › Supplementary Table 1.pdf]

Supplementary Table S1. Summary of time course experiments 1-4 to determine the duration of *Colpodella* sp.ATCC 50594) life cycle.

Time Point	Description
T0= 0 hours	Had a few *Parabodo* cysts and young *Colpodella* sp. cysts
T1= 4 hours	A few *Parabodo* trophozoites
T2= 8 hours	A little more *Parabodo* trophozoites
T3= 12 hours	Many *Parabodo* trophozoites
T4= 16 hours	Lots of *Parabodo* trophozoites
T5= 20 hours	Many *Bodo* and a few *Colpodella* trophozoites
T6= 24 hours	More *Colpodella* sp. trophozoites and first attachments
T7= 28 hours	*Colpodella* sp. display very long attachments and *Parabodo* cysts starting to form, both species have many trophozoites
T8= 32 hours	Some attachments and more *Parabodo* cysts forming, both species have many trophozoites
T9= 36 hours	Many *Parabodo* cyst, *Colpodella* sp. are fewer in number
T10= 5 days	Identified *Parabodo* cysts
T11= 10 days	Many *Parabodo* cysts
