# Supplementary material for: Colpodella sp. (ATCC 50594) Life Cycle: Myzocytosis and Possible Links to the Origin of Intracellular Parasitism
Source: Trop Med Infect Dis. 2021 Jul 11;6(3):127. doi: 10.3390/tropicalmed6030127 (PMC8293349; doi:10.3390/tropicalmed6030127)
Supplement: Supplementary file 1 [file tropicalmed-06-00127-s001.zip › Supplementary Table 2.pdf]

Supplementary Table S2. Time Course five observations during the most active parts of the *Colpodella* sp. (ATCC 50594) life cycle. Time point numbers correspond to hours/days cells were collected for staining.

**Time Point**	**Description**
T0 = 0 hours	Had a few *Parabodo caudatus* cysts * (Need more sample)
T1 = 4 hours	A few *Parabodo caudatus* trophozoites and cysts
T2 = 8 hours	A little more *P. caudatus* trophozoites
T3 = 12 hours	More *P. caudatus* trophozoites and *Colpodella* sp. mature cyst
T4 = 16 hours	Lots of *P. caudatus* trophozoites and first attachment, Saw *Colpodella* sp. early and late cyst
T5 = 20 hours	Many *P. caudatus* trophozoites and *Colpodella* sp. late cysts
T6 = 22 hours	Lots of *P. caudatus* trophozoites and some *Colpodella* sp. trophozoites, saw both early and mature cysts
T7 = 24 hours	*P. caudatus* trophozoites *and Colpodella* sp. attachments, early Colpodella cyst (2), mature Colpodella cyst (4)
T8 = 25 hours	*P. caudatus* trophozoites, many attachments, and saw a few *Colpodella* sp. early cysts
T9 = 26 hours	*P. caudatus* trophozoites and *Colpodella* sp. attachments, many early *Colpodella* sp. cysts, *P. caudatus* start encysting
T10 = 27 hours	*P. caudatus* trophozoites and cysts, some *Colpodella* sp. trophozoites with attachments, seeing *Colpodella* sp. encyst and early cysts
T11 = 28 hours	*P. caudatus* trophozoites and cysts, *Colpodella* sp. trophozoites and attachments, Most mature *Colpodella* sp. cysts
T12 = 29 hours	*P. caudatus* trophozoites and cysts, *Colpodella* sp. trophozoites and early cysts
T13 = 30 hours	More *P. caudatus* cysts than trophozoites, many *Colpodella* sp. attachments and saw some early cysts
T14 = 32 hours	*P. caudatus* cysts and trophozoites, *Colpodella* sp. trophozoites with a few attachments, a few early and late cysts
T15 = 34 hours	*P. caudatus* cysts with a few trophozoites, one *Colpodella sp*. attachment, saw early *Colpodella* sp. cyst
T16 = 36 hours	*P. caudatus* cysts and a few *Colpodella* sp. trophozoites
T17 = 38 hours	*P. caudatus* cysts, saw a few *Colpodella* sp. trophozoites and mature cysts
T18 = 40 hours	*P. caudatus* cysts
T19 = 5 days	A few *P. caudatus* cysts, saw *Colpodella* sp. mature cyst
T20 = 7 days	A few *P. caudatus* cysts
