# Supplementary material for: Colpodella sp. (ATCC 50594) Life Cycle: Myzocytosis and Possible Links to the Origin of Intracellular Parasitism
Source: Trop Med Infect Dis. 2021 Jul 11;6(3):127. doi: 10.3390/tropicalmed6030127 (PMC8293349; doi:10.3390/tropicalmed6030127)
Supplement: Supplementary file 1 [file tropicalmed-06-00127-s001.zip › Supplementary Table 3.pdf]

Supplementary Table S3.

Species	Method of Obtaining	Prey/Food	Medium of Growth	Fixation/Suspension/ DNA Extraction	Observation Method
[a] *Colpodella pseudoedax* & [b] *Colpodella unguis*	Institute of Biology of Inland Waters, Russian Academy of Science	Bacteriotrophic flagellates *Spumella* sp. *Procryptobia sorokini*	Pratt medium and Schmalz-Pratt medium	Centrifuged and fixed in mixture of 2% OsO4 and 0.6% Glutaraldehyde On Schmalz-Pratt media for 15-30min at 1C. A series of dehydrations occur in alcohols and anhydrous acetone, then placed in Araldite and Epons resins	First used BIOLAM-I microscope with phase contrast device KF-5 in passing light and water immersion. Second, used transmission electron microscope on thin sections
[c] *Colpodella gonderi*	Upland grassland site at Sourhope research station of Macaulay Land Use Research Institute, land was mid-altitude and temperate, shallow brown soil with poor minerals, pH at 4.5-4.8 taken at a depth of 5 cm, transported in 4C dark boxes to lab	*Colpoda steinii, Grossglockneria acuta, Psuedoplatyophyra nana*	Soil spread evenly in 15cm Petri dishes at room temp for 6 days, soil then passed through 3.35 sieve and homogenized, 5g air dried soil and 15 ml rainwater mixed into petri dish and incubated at 15C for 4 days	50 microliters of runoff put in Sedgewick-Rafter counting chamber and observed, ciliates identified by silver impregnation techniques (pyridinated silver carbonate methods)	Sedgewick-Rafter counting chamber, electron microscopy
[d] *Colpodella vorax*	Pond water that had dead leaves and grass	*Bodo caudatus, Spumella* sp. *Synura petersenii, Chilomonas paramaecium, Euglena gracilis, Colpoda cucullus*	Multiplied in pond water, Petri dish	Predator phase collected with pipette using a stereomicroscope, cells centrifuged and fixed by 1 vol of 1% glutaraldehyde in 0.1 M cacodylate buffer pH 7 and 1 vol of 1% osmium tetroxide for 1 hour, wash in distilled water cells are pre-embedded in 1% agar contrasted "en bloc" by saturated uranyl acetate in 50% ethanol then dehydrated in alcohol series, final embedding in Epon 812 resin	Using small chambers with ring of Vaseline on slide covered by slip C. vorax and a prey are photographed for hours/days with phase contrast microscopy, sections from Reichert Ultracut S microtome contrasted by lead citrate for 15 min and coated with carbon for EM

Species	Method of Obtaining	Prey/Food	Medium of Growth	Fixation/Suspension/ DNA Extraction	Observation Method
[e] *Voromonas pontica*	1)Isolated from coastal waters of Black Sea near Yalta, Institute of Biology of Inland Waters, Russian Academy of Science	*Escherichia coli*, *Bodo sorokini*	Schmaltz-Pratt medium (1L H2O, 28.15g NaCl,0.67g KCl, 5.51g MgCl26H2O, 6.92g MgSO47H2O, 1.45g CaCl2H2O, 0.1g KNO3, 0.01g K2HPO43H2O), 20-22% salinity	DNA extracted from pellets using hexadecyltri-methylammonium bromide	small subunit rRNA purified amplified with primers and PCR and seen on agrose gel, products isolated with ultraclean purification kit and inserted in 1) pGEM-T vector and 2) pCR 2.1 vector, each was sequenced with ABI big dye reaction mix using primers
[f] *Colpodella pugnax*	Artificial hypersaline (26% NaCl) lagoon at Whyalla, South Australia	*Dunaliella viridis*	28C, lighting conditions varied	Centrifuged and fixed for 30 mins in solution of 5% glutaraldehyde and 1% osmium tetroxide in a buffer of 18% NaCl, 110 HEPES, and 20 mM EDTA. Then wash in half strength buffer embedded in 2% agar and stained with saturated uranyl acetate in 50% ethanol for 2 hours, after blocks are dehydrated through ethanol series then embedded in Araldite resin	Light microscopy, Photomicrography, serial sections cut and collected on pioloform-coated slot grids and coated with carbon then stained with lead citrate for EM
[g] *Colpodella angusta*	Unknown	*Procryptobia soronki*, *Spumella sp.*, *Parabodo caudatus*	Unknown	Centrifugation and total RNA was reversed transcribed using SMARTer Pico PCR cDNA kit, DNA extraction kit, Phylogenetic research, GenBank	PCR, phylogenetic trees

Species	Method of Obtaining	Prey/Food	Medium of Growth	Fixation/Suspension/ DNA Extraction	Observation Method
[h] *Chromera velia*	First isolated from stony coral *Pleslastrea versipora* in Australia, this was obtained from Boothbay Harbor, ME from the culture collection of marine phytoplankton	*Procryptobia soronki, Spumella sp., Parabodo caudatus*	Cultivated in f/2 medium in sea water with a 12-hour light and dark cycle at 26 degrees with a 3.15 Watt intensity light	Cells could be fixed with glutaraldehyde and then pelleted and post fixed with OsO4 and dehydrated in acetone. Pellets were frozen using a high-pressure freezer and frozen substitution with liquid nitrogen. The temperature was slowly raised over a period of time and the cells were washed with acetone and resin. They were occasionally microwaved to allow the resin to polymerize. Sections were cut and stained with uranyl acetate and lead citrate and then carbon coated for TEM.	Light microscopy, electron microscopy, transmission electron microscopy, to look at morphology and ultrastructure.
[i] *Vitrella brassicaformis*	Isolated from One Tree Island, the Great Barrier Reef from a stony coral *Leptastrea purpurea*	*Procryptobia soronki, Spumella sp., Parabodo caudatus*	f/2 medium in saltwater at 22 degrees	Centrifuged for pellets and mixed with lysis buffer, RNA extracted using RNA kit	Scanning electron microscopy, Light microscopy, transmission electron microscopy
[j] *Colpodella sp.* (ATCC 50594)	American Type Culture collection (Manassas, VA)	*Bodo caudatus, Klebsiella pneumoniae*	ATCC culture medium 802 (Sonneborn's Paramecium), Hay medium	rRNA extraction for phylogenetic tree relationship, DNA extracted for sequencing and PCR, cells pelleted and fixed for staining	Transmission electron microscopy, light microscopy, confocal light microscopy
[k] *Colpodella tetrahymenae*	Rainforest soil from La Selva, Costa Rica	*Tetrahymena pyriformis*	Soil with natural mix of bacteria	DNA isolation and amplification, Phylogenetic analysis	Scanning electron microscopy, Light microscopy, transmission electron microscopy
