# Supplementary figures and images for: Colpodella sp. (ATCC 50594) Life Cycle: Myzocytosis and Possible Links to the Origin of Intracellular Parasitism
Source: Trop Med Infect Dis. 2021 Jul 11;6(3):127. doi: 10.3390/tropicalmed6030127 (PMC8293349; doi:10.3390/tropicalmed6030127)

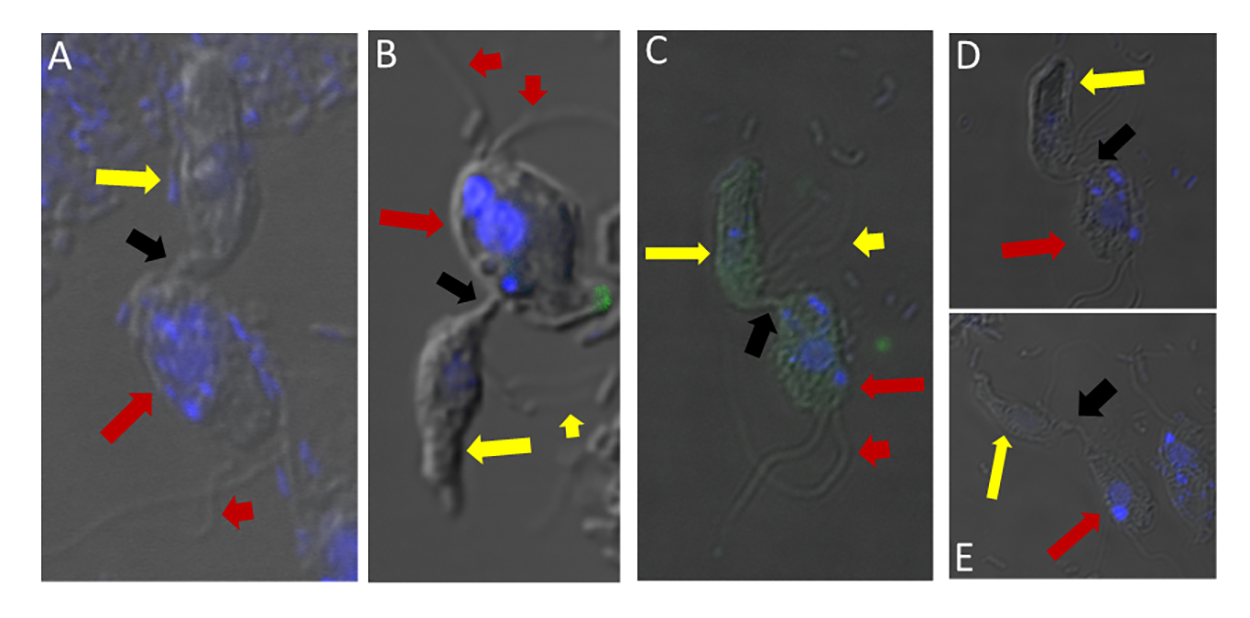

Supplement: Supplementary file 1 [file tropicalmed-06-00127-s001.zip › Supplementary Figure 1.tif]

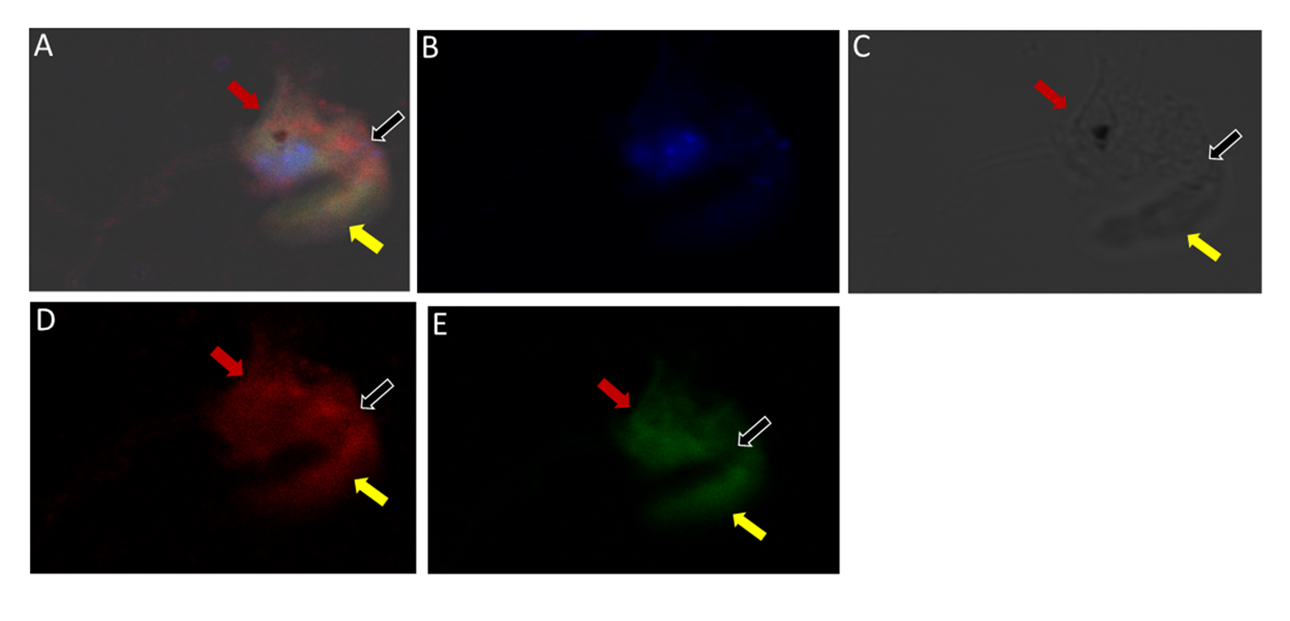

Supplement: Supplementary file 1 [file tropicalmed-06-00127-s001.zip › Supplementary Figure 3.tif]

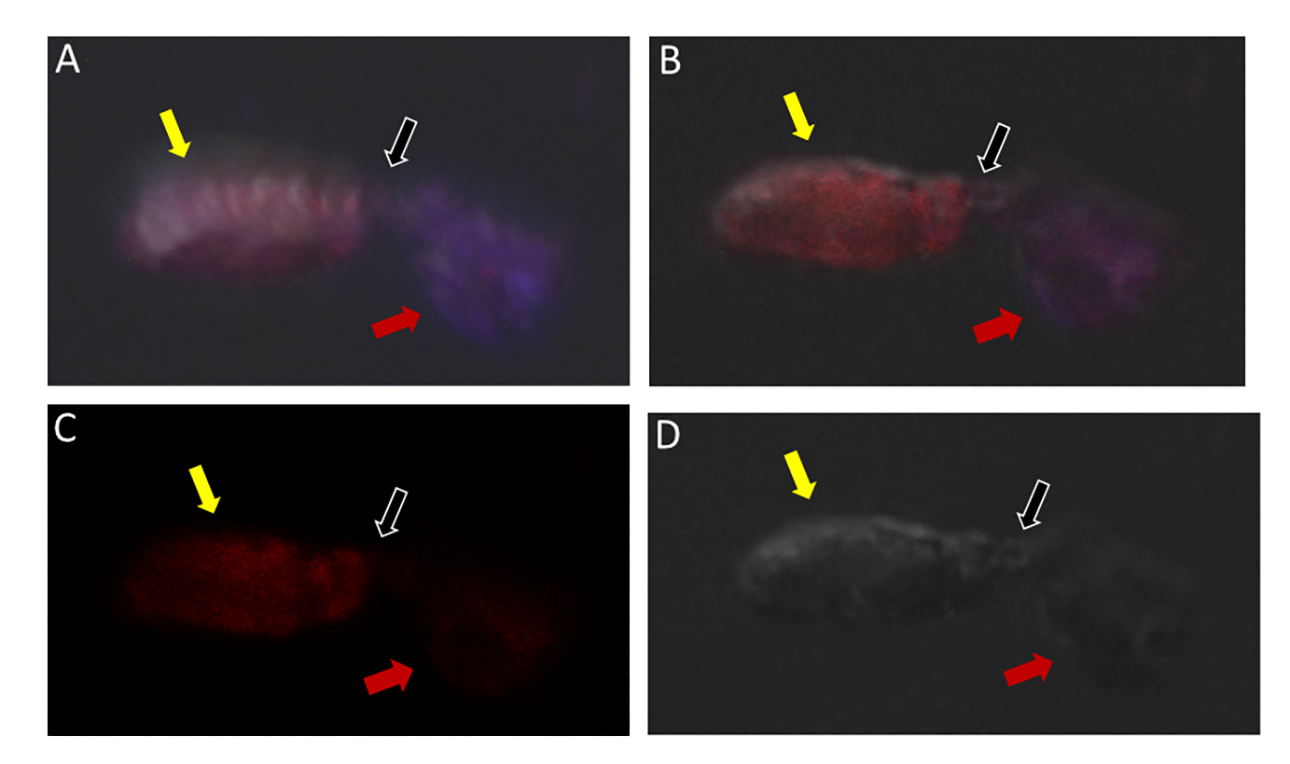

Supplement: Supplementary file 1 [file tropicalmed-06-00127-s001.zip › Supplementary Figure 4.tif]

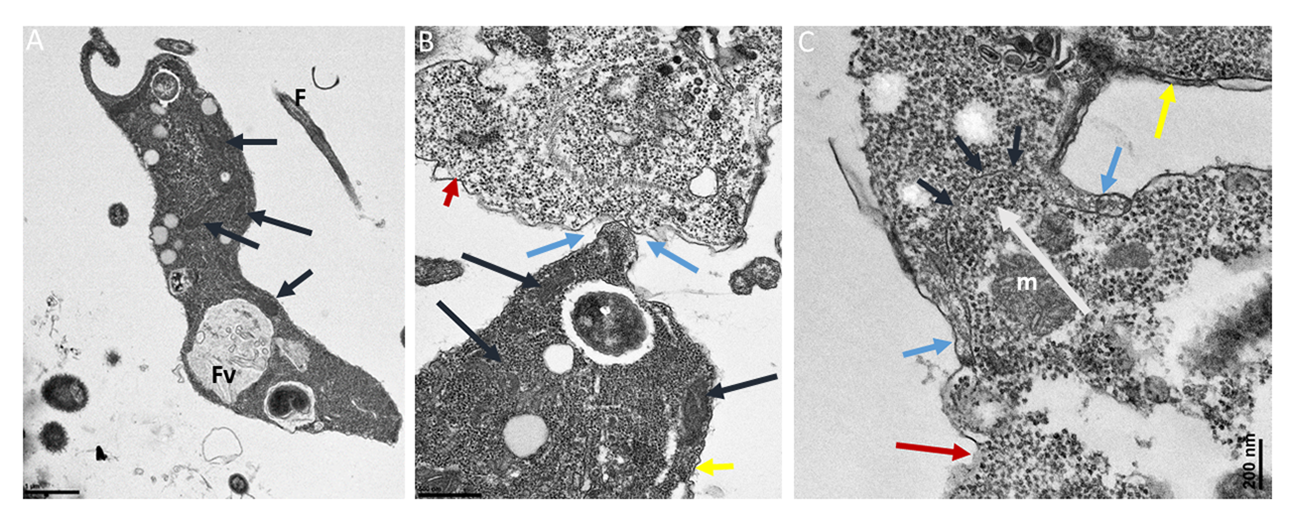

Supplement: Supplementary file 1 [file tropicalmed-06-00127-s001.zip › Supplementary Figure 6.tif]

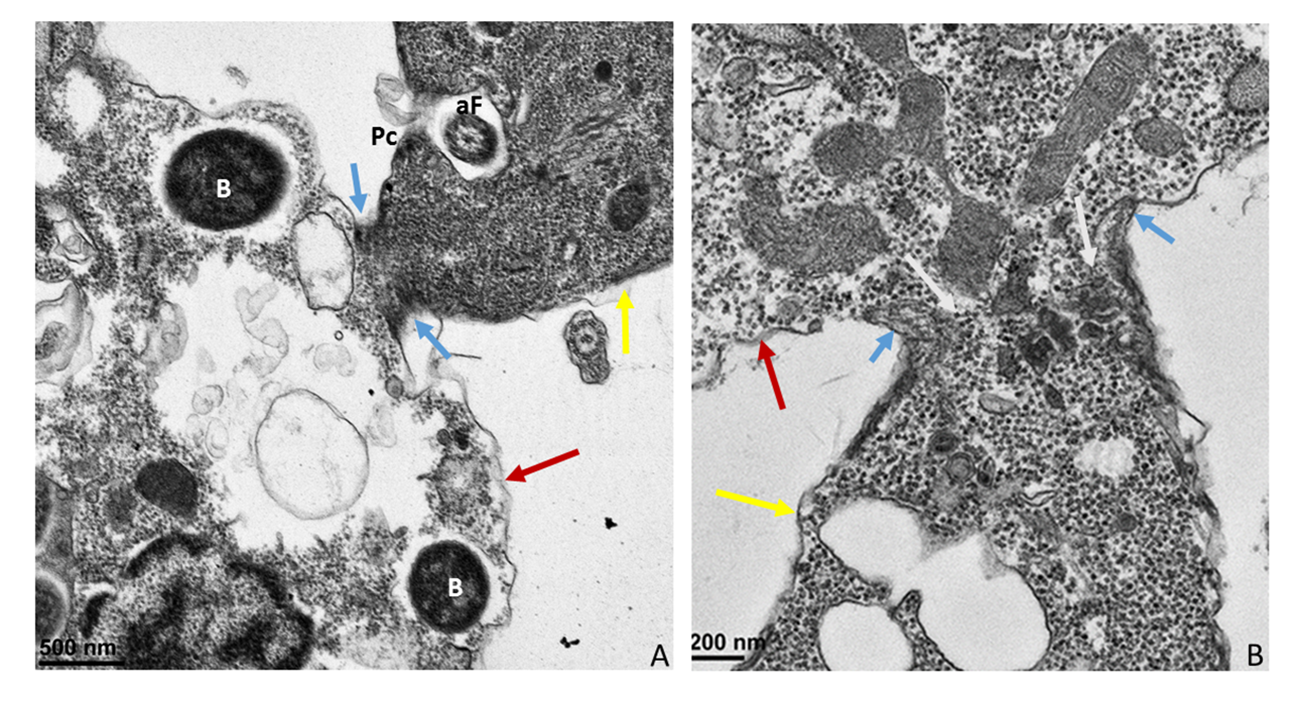

Supplement: Supplementary file 1 [file tropicalmed-06-00127-s001.zip › Supplementary Figure 7.tif]
